# Supplementary material for: Correction of pathology in mice displaying Gaucher disease type 1 by a clinically-applicable lentiviral vector
Source: Mol Ther Methods Clin Dev. 2020 Dec 3;20:312–23. doi: 10.1016/j.omtm.2020.11.018 (PMC7806948; doi:10.1016/j.omtm.2020.11.018)
Supplement: Document S1. Figures S1–S6 and Table S1 [file mmc1.pdf]

OMTM, Volume *20*

Supplemental Information

Correction of pathology in mice displaying Gaucher disease type 1 by a clinically-applicable lentiviral vector

Maria Dahl, Emma M.K. Smith, Sarah Warsi, Michael Rothe, Maria J. Ferraz, Johannes M.F.G. Aerts, Azadeh Golipour, Claudia Harper, Richard Pfeifer, Daniella Pizzurro, Axel Schambach, Chris Mason, and Stefan Karlsson

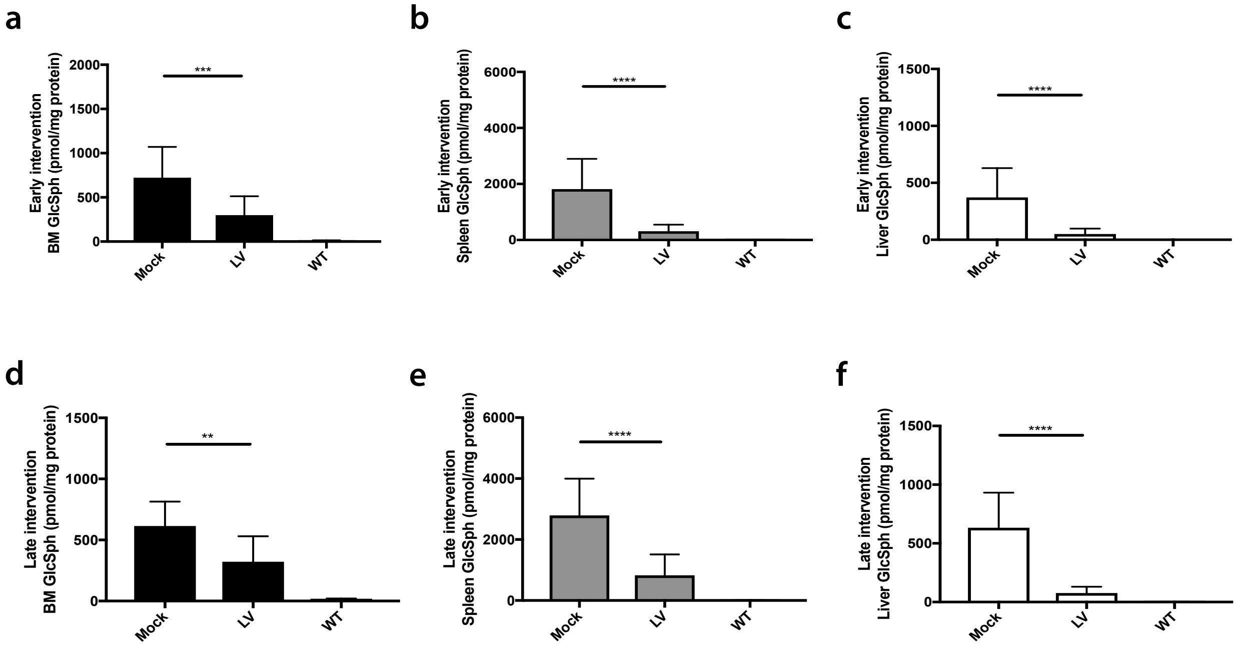

Fig S1) Drastically lowered Glucosylsphingosine accumulation in hematopoietic tissues after gene therapy.

Glucosylsphingosine (GlcSph) content was analyzed in bone marrow (BM), spleen and liver of mice from the Early and Late intervention study. Early intervention study (**a-c**); Mock, *n=13*; LV, *n=13*; WT, *n=8*. Late intervention study (**d-f**); Mock, *n=15*; LV, *n=10*; WT, *n=8*). Mann-Whitney *U*-test; *p = ≤ 0.05; **p = ≤ 0.01; ***p = ≤ 0.001; **** = p = ≤ 0.0001. Error bars represent mean + SD.

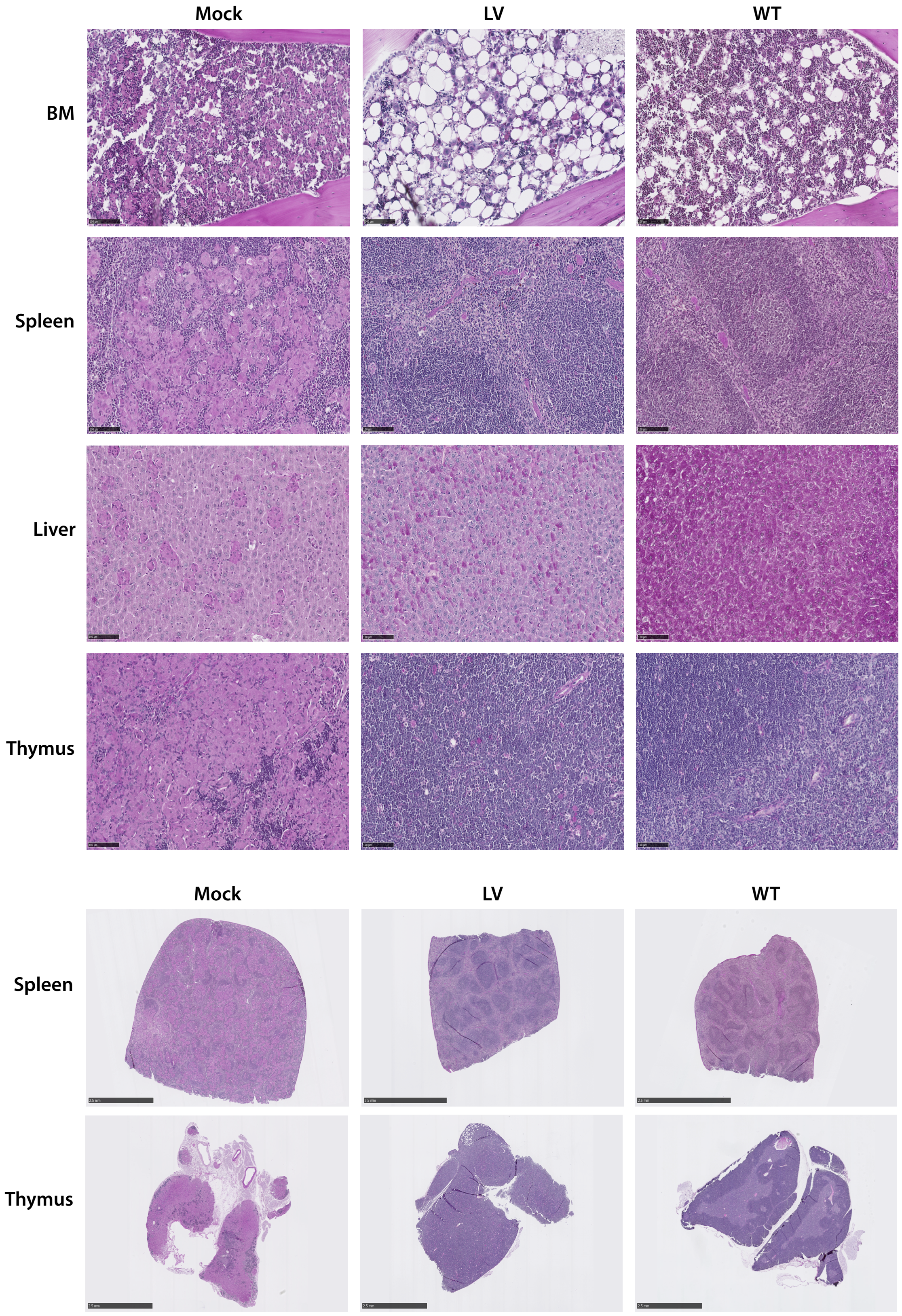

Fig S2) Representative samples of Gaucher cell infiltration in males of Early intervention study.

Periodic Acid Schiff staining of BM, spleen, liver and thymus samples from representative Mock, LV and WT male mice in the Early intervention study. Upper panel of BM, spleen, liver and thymus samples; black bars in lower left side represents scale bars of 100 μm. Lower panel of spleen and thymus tissue architecture; black bars in lower left side represents scale bars of 2.5 mm.

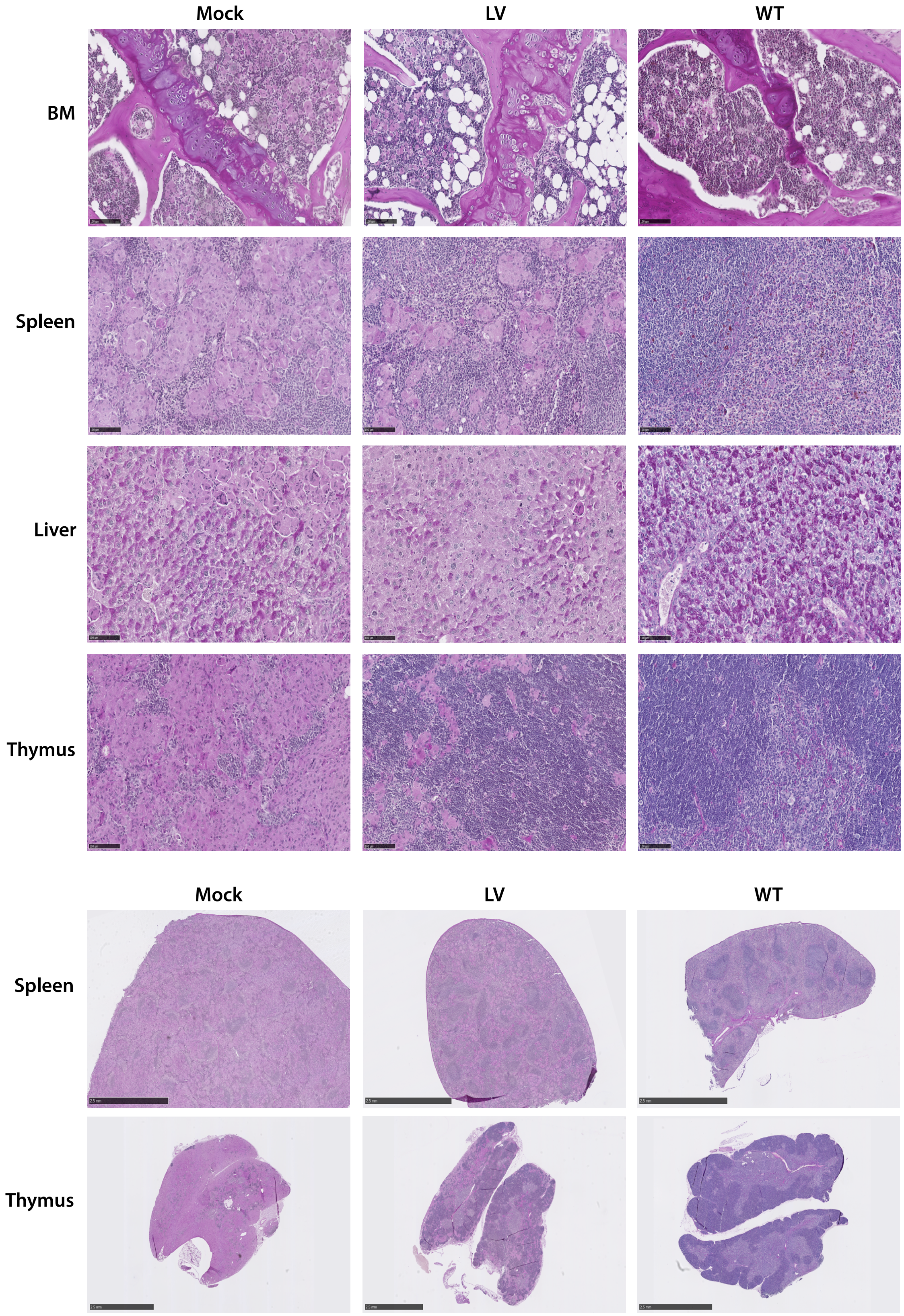

Fig S3) Representative samples of Gaucher cell infiltration in females of Late intervention study.

Periodic Acid Schiff staining of BM, spleen, liver and thymus samples from representative Mock, LV and WT female mice in the Late intervention study. Upper panel of BM, spleen, liver and thymus samples; black bars in lower left side represents scale bars of 100 µm. Lower panel of spleen and thymus tissue architecture; black bars in lower left side represents scale bars of 2.5 mm.

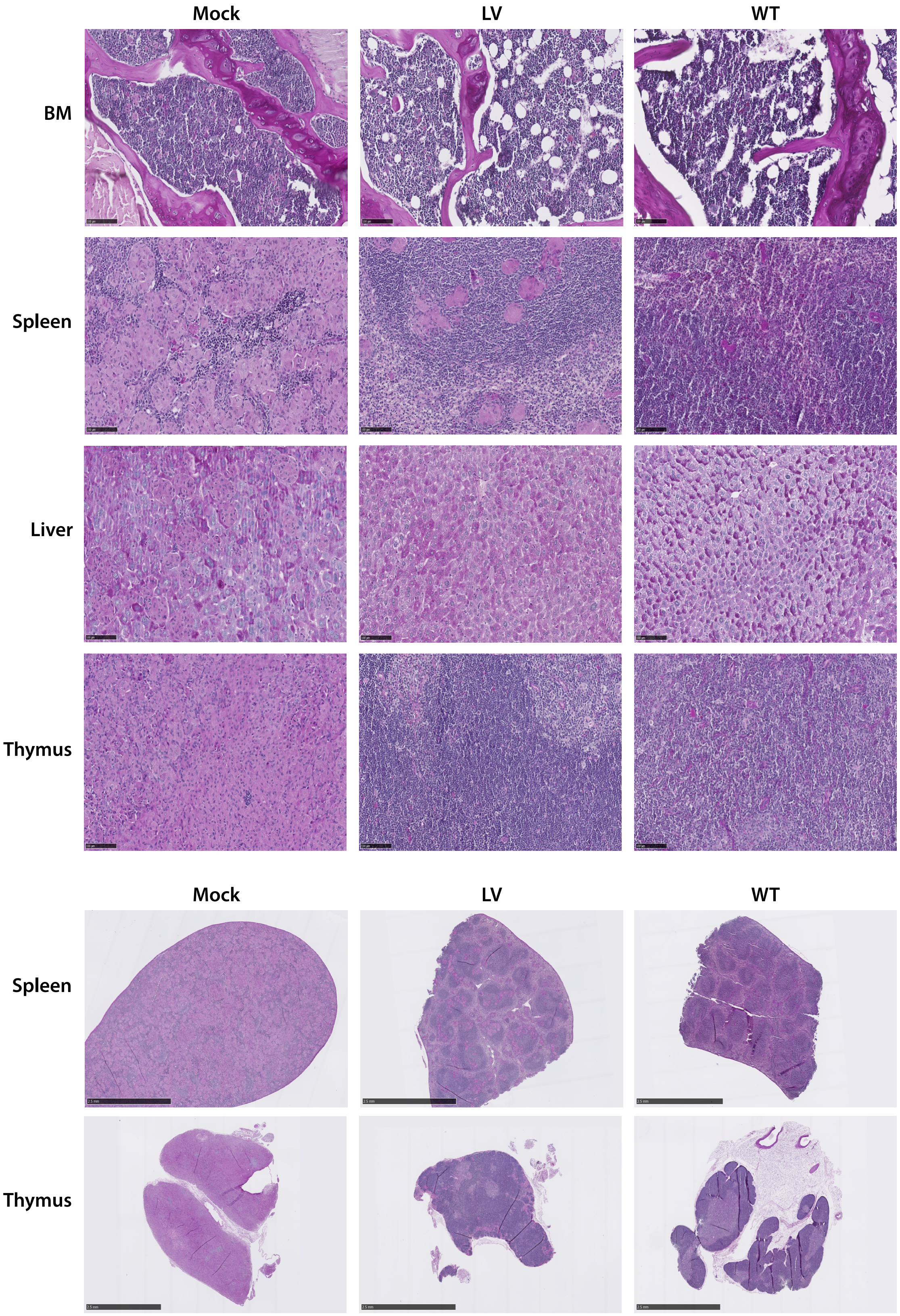

Fig S4) Representative samples of Gaucher cell infiltration in males of Late intervention study.

Periodic Acid Schiff staining of BM, spleen, liver and thymus samples from representative Mock, LV and WT male mice in the Late intervention study. Upper panel of BM, spleen, liver and thymus samples; black bars in lower left side represents scale bars of 100 μm. Lower panel of spleen and thymus tissue architecture; black bars in lower left side represents scale bars of 2.5 mm.

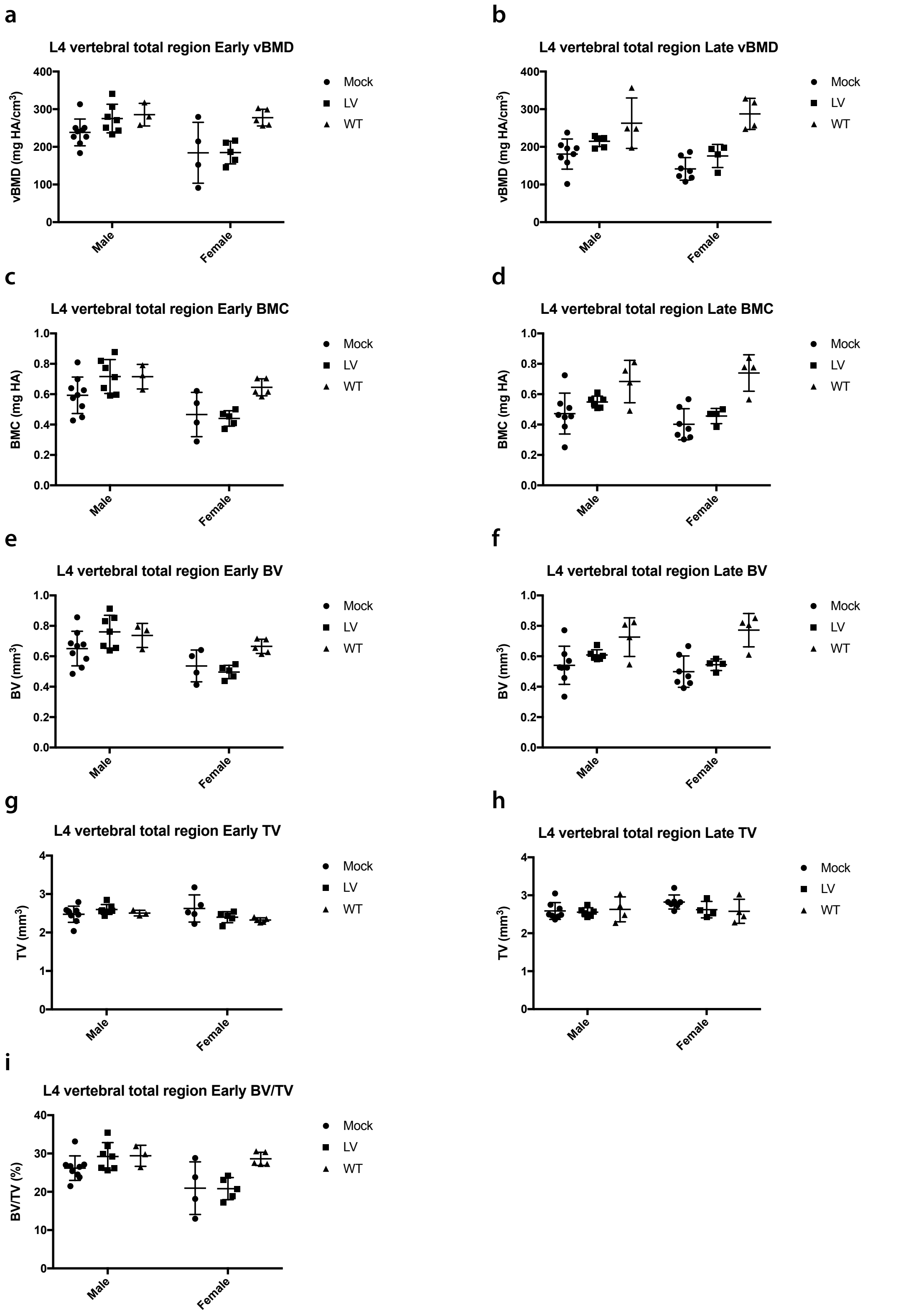

Fig S5) Micro-CT results from L4 vertebral region in male and female mice of Early and Late intervention study.

vBMD=volumetric bone mineral density, BMC=bone mineral content, BV=bone volume, TV=total volume, BV/TV=bone volume density. Early intervention samples in column to the left. Late intervention samples in column to the right.

Early intervention study males; Mock, *n=9*; LV, *n=8*; WT, *n=3*. Early intervention study females; Mock, *n=4*; LV, *n=5*; WT, *n=5*. Late intervention study males; Mock, *n=8*; LV, *n=6*; WT, *n=4*. Late intervention study females; Mock, *n=7*; LV, *n=4*; WT, *n=4*. Mann-Whitney *U*-test *p = ≤ 0.05; **p = ≤ 0.01; ***p = ≤ 0.001; **** = p = ≤ 0.0001. Error bars represent mean ± SD.

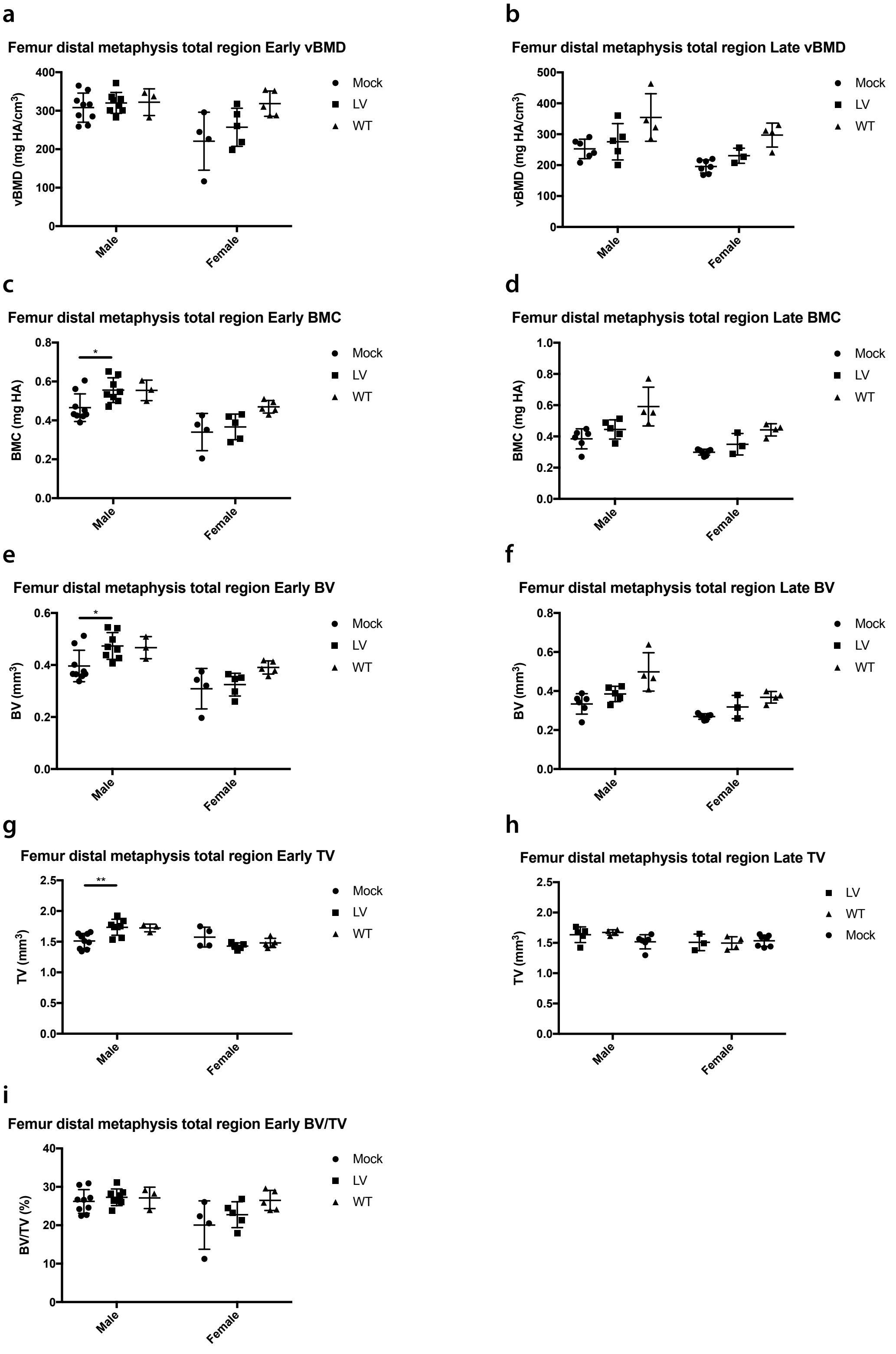

Fig S6) Micro-CT results from femur distal metaphysis region in male and female mice of Early and Late intervention study.

vBMD=volumetric bone mineral density, BMC=bone mineral content, BV=bone volume, TV=total volume, BV/TV=bone volume density. Early intervention samples in column to the left. Late intervention samples in column to the right.

Early intervention study males; Mock, *n=9*; LV, *n=8*; WT, *n=3*. Early intervention study females; Mock, *n=4*; LV, *n=5*; WT, *n=5*. Late intervention study males; Mock, *n=6*; LV, *n=5*; WT, *n=4*. Late intervention study females; Mock, *n=7*; LV, *n=3*; WT, *n=4.* Mann-Whitney *U*-test *p = ≤ 0.05; **p = ≤ 0.01; ***p = ≤ 0.001; **** = p = ≤ 0.0001. Error bars represent mean ± SD.

Table S1) Reoccurring insertions closest to individual genes (refseq_genes) between animals (overlaps) and for the *in vitro* control (multiple hit counts).

Between Animals	
**refseq_genes**	**overlap**
Lrrc4c	3
Ibtk	2

in vitro	
**refseq_genes**	**counts**
Lrrc4c	11
Mir101c	5
Tmem135	2
Celf4	2
Tmem114	2
Basp1	2
Lrtm1	2
Dcun1d5	2
Aplf	2
Ica1	2
Kit	2
Cdk5rap2	2
Fbxw7	2
Pcdh9	2
Loxl2	2
Arhgap10	2
