# Supplementary material for: Correction of pathology in mice displaying Gaucher disease type 1 by a clinically-applicable lentiviral vector
Source: Mol Ther Methods Clin Dev. 2020 Dec 3;20:312–23. doi: 10.1016/j.omtm.2020.11.018 (PMC7806948; doi:10.1016/j.omtm.2020.11.018)
Supplement: Document S2. Article plus Supplemental Information [file mmc2.pdf]

Molecular Therapy
Methods & Clinical Development
Original Article

Correction of pathology in mice displaying Gaucher disease type 1 by a clinically-applicable lentiviral vector

Maria Dahl,[1,7] Emma M.K. Smith,[1,7] Sarah Warsi,[1] Michael Rothe,[2] Maria J. Ferraz,[3] Johannes M.F.G. Aerts,[3] Azadeh Golipour,[4] Claudia Harper,[4] Richard Pfeifer,[4] Daniella Pizzurro,[4] Axel Schambach,[2,5] Chris Mason,[4,6] and Stefan Karlsson[1]

[1]Department of Molecular Medicine and Gene Therapy, Lund University, Lund, Sweden; [2]Hannover Medical School, Institute of Experimental Hematology, Hannover, Germany; [3]Department of Medical Biochemistry, Leiden University, Leiden, the Netherlands; [4]AVROBIO, Inc., Cambridge, MA, USA; [5]Division of Hematology/Oncology, Boston's Children's Hospital, Harvard Medical School, Boston, MA, USA; [6]University College London, Advanced Centre for Biochemical Engineering, London, UK

Gaucher disease type 1 (GD1) is an inherited lysosomal disorder with multisystemic effects in patients. Hallmark symptoms include hepatosplenomegaly, cytopenias, and bone disease with varying degrees of severity. Mutations in a single gene, glucosidase beta acid 1 (*GBA1*), are the underlying cause for the disorder, resulting in insufficient activity of the enzyme glucocerebrosidase, which in turn leads to a progressive accumulation of the lipid component glucocerebroside. In this study, we treat mice with signs consistent with GD1, with hematopoietic stem/progenitor cells transduced with a lentiviral vector containing an RNA transcript that, after reverse transcription, results in codon-optimized cDNA that, upon its integration into the genome encodes for functional human glucocerebrosidase. Five months after gene transfer, a highly significant reduction in glucocerebroside accumulation with subsequent reversal of hepatosplenomegaly, restoration of blood parameters, and a tendency of increased bone mass and density was evident in vector-treated mice compared to non-treated controls. Furthermore, histopathology revealed a prominent reduction of Gaucher cell infiltration after gene therapy. The vector displayed an oligoclonal distribution pattern but with no sign of vector-induced clonal dominance and a typical lentiviral vector integration profile. Cumulatively, our findings support the initiation of the first clinical trial for GD1 using the lentiviral vector described here.

INTRODUCTION

Gaucher disease (GD) belongs to the family of lysosomal disorders and is inherited in an autosomal recessive manner.[1,2] The disorder may present at any age from early childhood to old age and symptom presentation and burden vary significantly between patients.[3] GD has an incidence of one in 40,000–60,000 live births, although it is more frequent in certain populations such as the Ashkenazi Jews (one in 800 live births).[2,3] GD is a monogenic disorder caused by insufficient activity of the enzyme glucocerebrosidase (also known as glucosylceramidase [GCase]). Mutations of the glucosidase beta acid 1 (*GBA1*) gene are the underlying cause of Gaucher disease. All mutations give rise to an enzyme product with decreased, and in severe cases complete lack of, activity. An inherited deficiency of functional GCase causes a progressive build-up of the lipid component glucocerebroside (glucosylceramide [GlcCer]), primarily in lysosomes of mononuclear phagocytic cells (macrophages). With a progressive accumulation of GlcCer, disease-characteristic "Gaucher cells" appear. These are typically detected under the light microscope as enlarged cells with eccentric nuclei.[3]

GD is commonly divided into three clinical subtypes depending on the presence and severity of central nervous system (CNS) symptoms.[2,4] Gaucher disease type 1 (GD1) is associated with minor or no neurological manifestations and is the most common form, constituting 94% of all cases in the Western world.[3,5] Although GD1 is seldom life threatening, it often hampers quality of life and is associated with considerable morbidity. Splenomegaly is observed in over 90% of patients and spleen sizes can be massive, weighing several kilograms and causing abdominal discomfort or pain.[6] Hepatomegaly is detected in 60%–80% of patients.[7] Thrombocytopenia is diagnosed in 60%–90% of patients while anemia is less frequent (20%–50% of cases) and leukopenia is rare.[3,8] Bone disease is a debilitating symptom of GD and can give rise to acute and painful bone crisis events.[9,10] A point of note is that *GBA1* mutations are the most common genetic risk factor for Parkinson's disease.[11]

For decades, the gold standard treatment for GD type 1 patients has been enzyme replacement therapy (ERT), involving intravenous

Received 10 August 2020; accepted 28 November 2020;
https://doi.org/10.1016/j.omtm.2020.11.018.

[7]These authors contributed equally

Correspondence: Stefan Karlsson, Department of Molecular Medicine and Gene Therapy, Lund University, Lund, Sweden.
E-mail: stefan.karlsson@med.lu.se

 © 2020 The Authors.
This is an open access article under the CC BY license (http://creativecommons.org/licenses/by/4.0/).

infusions of recombinant-produced GCase twice a month. Another treatment option is an oral substrate reduction therapy (SRT), reducing the synthesis of GlcCer. Although ERT and SRT can be effective in alleviating disease symptoms such as hepatosplenomegaly and improving blood parameters, it requires patients to continue drug administration for the remainder of their lives and has limited effect on the debilitating bone disease.[12,13] Currently, the only potentially curative option for GD1 is transplanting allogeneic hematopoietic stem and progenitor cells (HSPCs) from a healthy and immune compatible donor to the patient. However, there are major disadvantages associated with this type of treatment due to the requirement in allografting for immunosuppressive conditioning (e.g., busulfan and cyclophosphamide), as well as specific challenges associated with allografts including the risk of graft-versus-host disease.[14] Autologous HSPC transplants typically only require single agent myeloid conditioning, such as busulfan, in order to create the required space in the bone marrow. *Ex vivo* gene therapy, being autologous-based, offers the potential of being a curative approach while avoiding the side effects associated with polypharmacy immunosuppressive conditioning and allogeneic HSPC transplantation (allo-HSCT). There are currently more than 350 patients, with more than 10 different monogenetic disorders, who have undergone *ex vivo* genetic correction of autologous HSPCs using lentiviral vectors as recently reviewed by Cavazzana and others.[15,16] To date, no serious adverse events (SAEs) due to insertional mutagenesis have been reported for these patients.[15] Clinical trials using lentiviral vectors for treating the metabolic disorders cerebral adrenoleukodystrophy (CALD) (ClinicalTrials.gov: NCT01896102) and metachromatic leukodystrophy (MLD) (ClinicalTrials.gov: NCT01560182) are ongoing with highly beneficial results to date.[17–20] Previous proof-of-concept studies using retroviral and lentiviral vectors have been successful in treating hallmark GD1 signs in our murine disease model.[21,22] In this paper, we show that a single gene self-inactivating (SIN) lentiviral vector enabling the integration of human glucocerebrosidase gene under the control of a physiological promoter into hematopoietic stem/progenitor cells, may support a safe and efficacious therapy for treating GD1. These data provided the necessary proof-of-concept for a successful regulatory filing in USA, Canada, and Australia and the ongoing phase 1/2 clinical trial ("Phase 1/2 Lentiviral Vector Gene Therapy - The GuardOne Trial of AVR-RD-02 for Subjects With Type 1 Gaucher Disease," ClinicalTrials.gov: NCT04145037).

RESULTS

The EFS.GBA SIN lentiviral vector stably integrates into hematopoietic stem- and progenitor cells

In this study, we aimed to evaluate whether a lentiviral vector containing an RNA transcript that, after reverse transcription, results in codon-optimized cDNA that, upon its integration into the human genome, encodes for functional human glucocerebrosidase under the control of the elongation factor 1α short (EFS) promoter, could halt Gaucher disease type 1 progression (early intervention study), or alternatively correct an already established disease state (late intervention study) (Figures 1A and 1B). For this purpose, we used a conditional murine disease model previously established in our laboratory. The Gaucher disease type 1 mice (henceforth referred to as GD1 mice) develop the classic manifestations of the disease such as increased spleen size with prominent Gaucher cell infiltration.[22] The same transduction and transplantation protocol was used in both intervention approaches. Briefly, the lineage negative (Lin^-) bone marrow compartment enriched with hematopoietic stem- and progenitor cells from donor GD1 animals was used for overnight transduction with the lentiviral vector (LV). The following day, LV-transduced or mock-transduced cells, respectively, were transplanted into fully ablated GD1 recipients and treatment efficacy evaluated 5 months later (Figure 1B).

As manifestations of the disease model develop progressively with the amount of GlcCer accumulated in mice, GD1 mice normally do not present with physical signs before 5 months post induced GBA1 exon deletion.[22] Thus, mice between 6 and 9 months of age were anticipated to demonstrate established Gaucher disease, i.e., pronounced splenomegaly and extensive Gaucher cell infiltration, and were therefore used in the Late intervention experiments. To evaluate whether a gene therapy approach is beneficial in a disease state where glucocerebrosidase activity is diminished, albeit mice present with mild clinical signs, we constructed experiments utilizing mice with increased substrate levels but devoid of severe physical signs. We refer to these experiments as the early intervention study. The rationale behind an early intervention approach is that it simulates the condition/state of patients on biweekly infusions of enzyme or who are presymptomatic (following early diagnosis), while a late intervention approach, in turn, would be more similar to a situation where the patient has chronically progressed without any conventional treatment such as ERT or SRT.

Initial transduction efficiencies were evaluated by quantitative real-time polymerase chain reaction (qPCR) 48 h post transduction. The number of vector copy number insertions per diploid genome (VCN/dg) in the transduced cell pools ranged between 3.1–3.3 for individual transductions carried out in the early intervention experiments and 2.1–3.2 in the late intervention experiments (Figure 1C). No vector backbone element could be detected in mock-transduced cells. Aliquots of cells from the transduced wells were seeded in semisolid media containing hematopoiesis-stimulating cytokines (colony-forming unit [CFU] assay). After 10–12 days of culture, individual hematopoietic colonies were picked and analyzed for the presence of vector backbone. In the early intervention experiments, vector positive colonies ranged between 70%–84% while in the late intervention experiments 36%–72% of colonies were found to have integrated vector (Figure 1D).

In summary, the EFS SIN LV-vector stably integrates into colony-forming hematopoietic stem-progenitor cells.

Efficient prevention of Gaucher disease progression in an early intervention approach

We investigated whether the EFS SIN LV-vector could halt disease progression in induced GD1 mice without established disease, (i.e.,

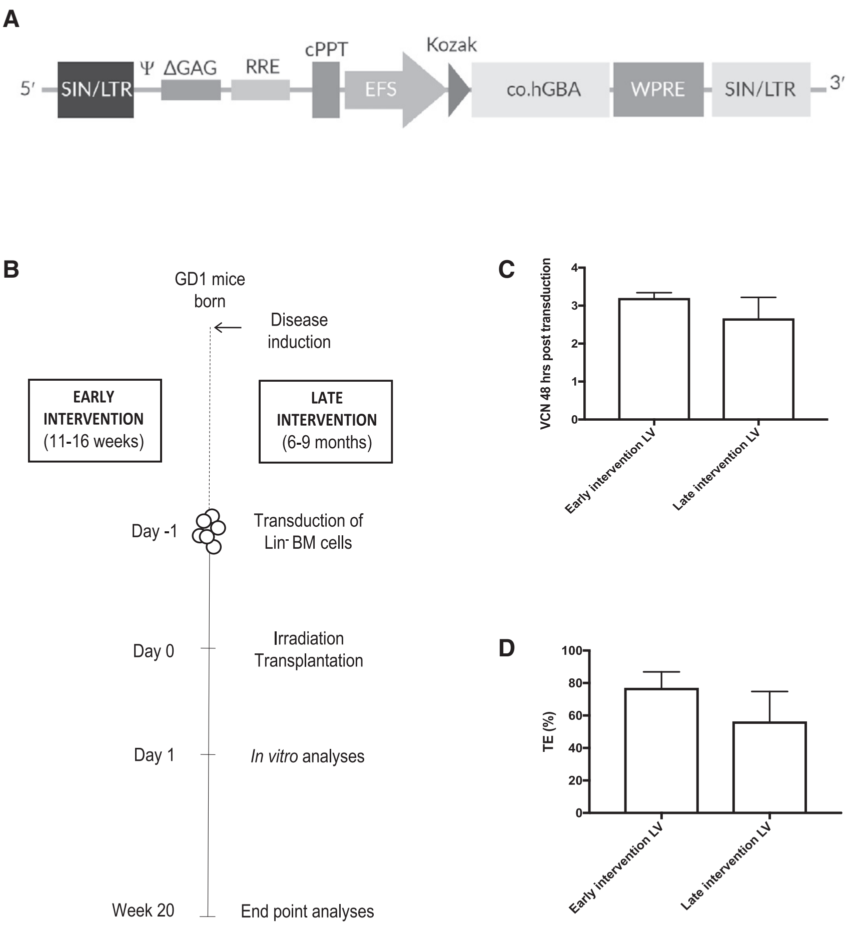

Figure 1. Schematic representation of EFS.GBA lentiviral vector integrated proviral DNA and experimental design

(A) The self-inactivating (SIN) lentiviral vector-integrated proviral DNA containing the human codon optimized glucocerebrosidase gene (*GBA*) cDNA driven by elongation factor 1 alpha short (EFS) promoter. (B) Two studies, the early and late intervention, were conducted using the conditional Gaucher mouse model (GD1 mice). GD1 mice were 11–16 weeks old at the start of the early intervention study while 6- to 9-month-old animals were utilized in the late intervention study. The same transduction and transplantation protocol were employed in both studies, lineage depleted (Lin^{-}) bone marrow (BM) cells were obtained from donor animals, transduced overnight, and transplanted the next day into lethally irradiated recipients. Analyses were conducted 20 weeks posttransplant. (C) Vector copy number (VCN) were estimated 48 h post transduction in a small aliquot of cells from each transduction by qPCR. (D) Transduced cells were used 48 h post transduction in colony forming unit (CFU) assay and single colonies analyzed by PCR after 10–12 days of culture. Transduction efficiency (TE) was estimated by PCR in 70–90 single colonies from each transduction experiment. Error bars represent mean + SD.

early intervention). Bone marrow (BM) from young mice (11–16 weeks of age) was obtained and used for transduction with subsequent transplantation to age-matched, lethally irradiated GD1 recipients (Figure 2A). At 20 weeks posttransplant, tissues from mock- and LV-transplanted mice were harvested and analyzed. Wild-type (WT) mice, of similar age and strain background as the GD1 mice, were used as a normal reference for all the biochemical and clinical parameters assessed. Mean VCN was analyzed in samples from hematopoietic organs. Average VCN levels were comparable between BM, blood, and spleen samples (ranging from 0.45–0.59 copies per cell). The lowest gene marking was observed in liver samples with an average VCN of 0.12 (Figure 2E). Mock animals did not have detectable vector in analyzed tissues. Quantification of GlcCer levels revealed significantly reduced lipid levels in BM, spleen, and liver of LV-treated animals compared to mock controls (Figures 2B–2D). In accordance with this, quantification of a minor substrate for GCase, namely glucosylsphingosine (GlcSph, the deactylated version of GlcCer), showed robustly decreased levels in the same tissues (Figure S1). Specifically, mean GlcCer levels were 79%–86% lower and mean GlcSph levels were 59%–86% lower in LV-treated mice compared to mock-treated across all tissues. Development of hepatosplenomegaly was efficiently prevented in animals treated with vector (Figures 2F and 2G). Furthermore, histopathological evaluation revealed that LV-treatment greatly reduced development of Gaucher cell infiltration while mock-treated mice had considerable amounts of infiltrating Gaucher cells in BM, spleen, liver, and thymus samples (Figure 4; Figure S2). Furthermore, spleen and thymus organ architecture was preserved in LV-treated mice, while this was disrupted in Gaucher cell-infiltrated mock animals (Figure 4; Figure S2).

In conclusion, an early intervention approach using the EFS.GBA SIN LV vector resulted in significantly reduced substrate levels, close to what is observed in WT animals, even by a relatively low level of gene transfer (on average <0.5 copies per cell). The therapeutic effect of the vector halted development of hepatosplenomegaly and suppressed Gaucher cell infiltration.

Robust reduction of established Gaucher disease pathology by lentiviral gene therapy

Next, we evaluated the efficacy of the vector in treating manifest Gaucher disease signs in late intervention experiments (Figure 3A). Average VCN was similar to what was observed in the early intervention experiments using the same transduction and transplantation protocol. The average copy number ranged between 0.43–0.74 in BM, blood, and spleen samples with the liver samples again showing the lowest gene marking with an average 0.16 copies per cell (Figure 3E). Mock mice did not have detectable vector in the examined tissues. The LV vector significantly decreased the substrate load in all tissues evaluated, but to a lesser degree in BM

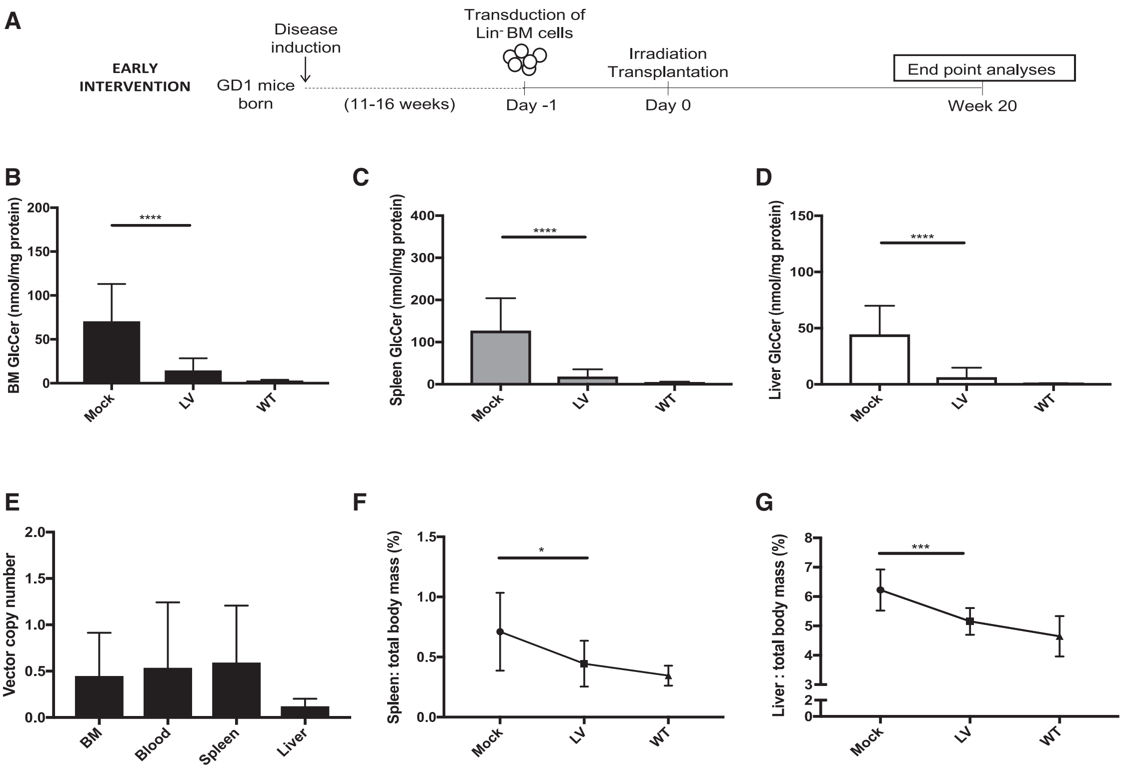

Figure 2. Efficient prevention of Gaucher disease in an early intervention approach

(A) Experimental design of the early intervention study. (B–D) Glucocerebroside (GlcCer) quantification in BM (B), spleen (C) and liver (D) 20 weeks posttransplant. (E) VCN was estimated in whole tissue lysates from BM, blood, spleen, and liver samples. (F) Relative spleen mass reduction (graph depicting the percentage of spleen mass: total body mass for individual mice). (G) Relative liver mass reduction (graph depicting the percentage of liver mass: total body mass for individual mice). Mock, n = 13; LV, n = 13; WT, n = 8. Mann-Whitney U-test; *p ≤ 0.05; **p ≤ 0.01; ***p ≤ 0.001; ****p ≤ 0.0001. Error bars represent mean ± SD.

than spleen and liver samples (Figures 3B–3D). Across tissues, mean GlcCer and GlcSph levels were 42%–83% and 48%–88% lower, respectively, compared to mock-recipient samples. Although there were quite large interindividual differences in terms of hepatosplenomegaly in the GD1 mice, treated animals exhibited a robust and significant decrease in spleen and liver mass compared to mock controls (Figures 3F and 3G). Previous characterization of the GD1 mouse model has revealed a mild form of microcytic anemia arising in the animals, albeit this is only noticeable in animals older than 1 year.[21,22] Neither leukopenia nor thrombocytopenia (an important clinical parameter in patients) are detectable in the animal model and are therefore not chosen as parameters for evaluating treatment efficacy. The red blood cell parameters in LV-treated mice showed robust improvement compared to mock in terms of hemoglobin (HGB) and hematocrit (HCT) levels (Figures 3H and 3I). There was also a trend of increased mean cell volume (MCV) in LV-treated animals, although this was not statistically significant (Figure 3J). As expected, no other differences in blood parameters were detected between mock and LV-treated mice (data not shown). Microscopic examination revealed a prominent decrease of infiltrating Gaucher cells in BM, spleen, liver, and thymus of treated mice compared to mock animals (Figures S3 and S4).

In summary, robustly reduced glucocerebroside levels, a dramatic reduction of spleen and liver mass and a restoration in certain red blood cell parameters was evident in mice 5 months after transplant of gene-corrected cells.

Tendencies of increased bone mass and density in gene-therapy-treated Gaucher mice

Skeletal involvement is a major cause of morbidity in Gaucher disease and varies widely between and within individual Gaucher disease patients.[23] We investigated whether a gene therapy approach would have beneficial effects on bone parameters in the GD1 mice. A micro-computed tomography (micro-CT) approach was applied to quantify differences in lumbar vertebrae and femur areas of transplanted animals. For proper comparisons of bone parameters, transplanted animals were divided into groups of male and female mice within each experimental cohort and intervention study. WT mouse samples were also analyzed, albeit comparisons to this steady-state group were not made as irradiation has been shown to result in loss of trabecular bone tissue with compromised microarchitecture in appendicular and axial skeleton of adult mice,[24–26] making irradiation a confounding factor when analyzing bone differences. However, just as for other datasets presented in this article,

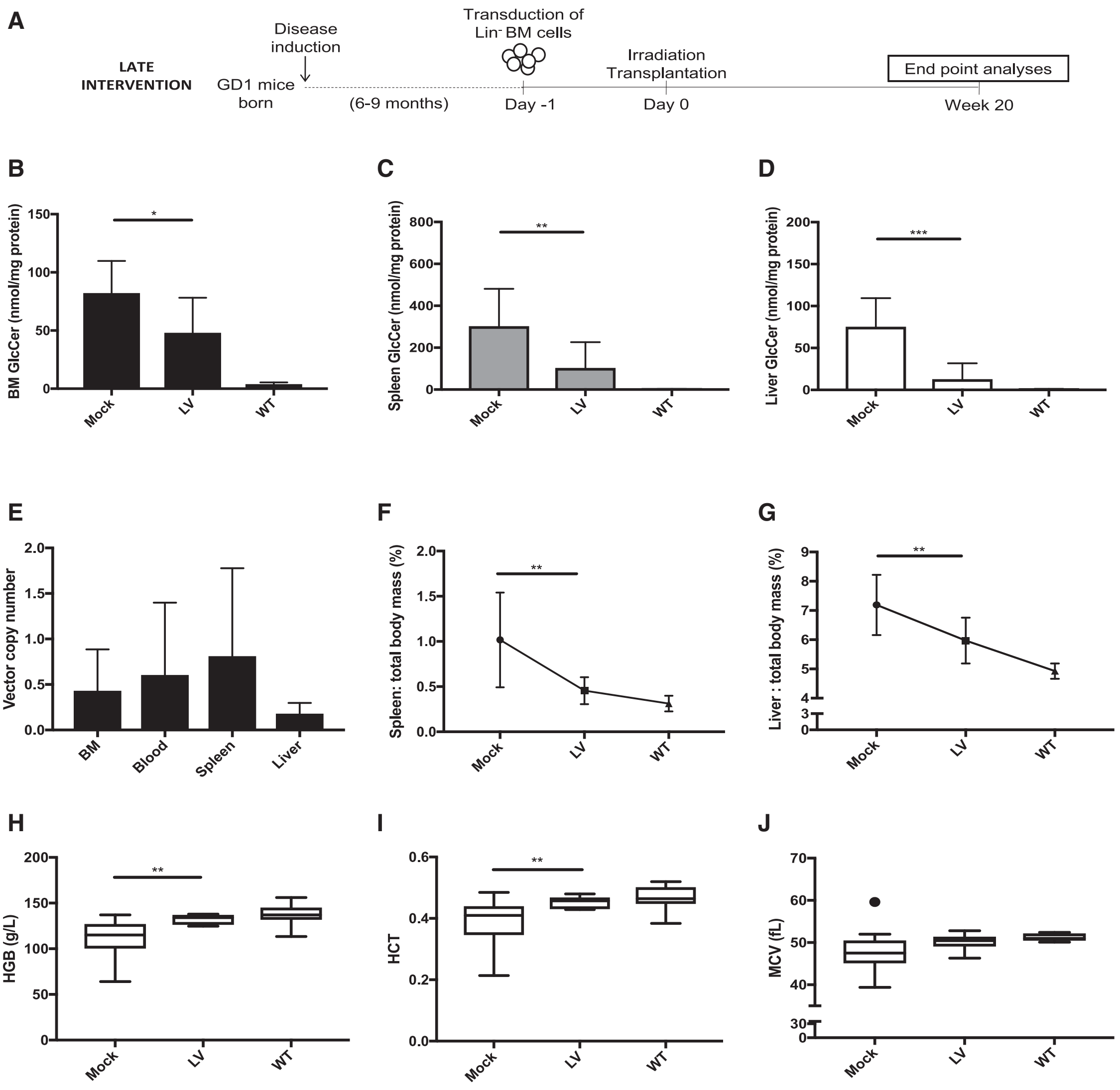

Figure 3. Robust reduction of established Gaucher disease pathology by lentiviral gene therapy

(A) Experimental design of the late intervention study. (B–D) GlcCer quantification in BM (B), spleen (C), and liver (D) 20 weeks posttransplant. (E) VCN was estimated in whole tissue lysates from BM, blood, spleen, and liver samples. (F) Relative spleen mass reduction (graph depicting the percentage of spleen mass: total body mass for individual mice). (G) Relative liver mass reduction (graph depicting the percentage of liver mass: total body mass for individual mice). (H–J) Blood samples analyzed for hemoglobin (HGB) (H), hematocrit (HCT) (I), and mean red blood cell volume (MCV) (J). Mock, n = 15; LV, n = 10; WT, n = 8. Mann-Whitney U-test; $^*p \leq 0.05$; $^{**}p \leq 0.01$; $^{***}p \leq 0.001$; $^{****}p \leq 0.0001$. Error bars represent mean ± SD.

WT samples were included to visualize the normal steady state of the different bone parameters.

Transplantation of LV-transduced cells in both males and females of the early intervention study resulted mainly in increases in bone mass and density, compared to mock cohorts, as assessed by volumetric bone mineral density (vBMD) and bone volume density (BV/TV) in L4 vertebrae and metaphysis region in femurs (Figures S5A, I and S6A, I). In LV-treated males of the early intervention cohort, the L4 assessed region presented an average increase of 15% in vBMD and 12% increase in BV/TV, along with on average 17% increase in BV and 21% in bone mineral content (BMC) compared to mock mice (Figures S5A, S5C, S5Ei). On average, female LV-transplanted mice showed no increase of these parameters compared to controls. In the late intervention cohorts, LV-transplanted male mice exhibited an average increase in vBMD and BV/TV of 19% and 15%, respectively, and a 13% increase of BV and 16% in BMC (Figure 5A; Figures S5B, S5D, and S5F). LV-transplanted females in the late intervention showed average increases of 24% in vBMD and 19% in BV/TV (Figure S5A; Figure 5A) along with 9% in BV and 14% in BMC compared to mock animals (Figures S5D and S5F). Similar tendencies were observed in the metaphysis region of

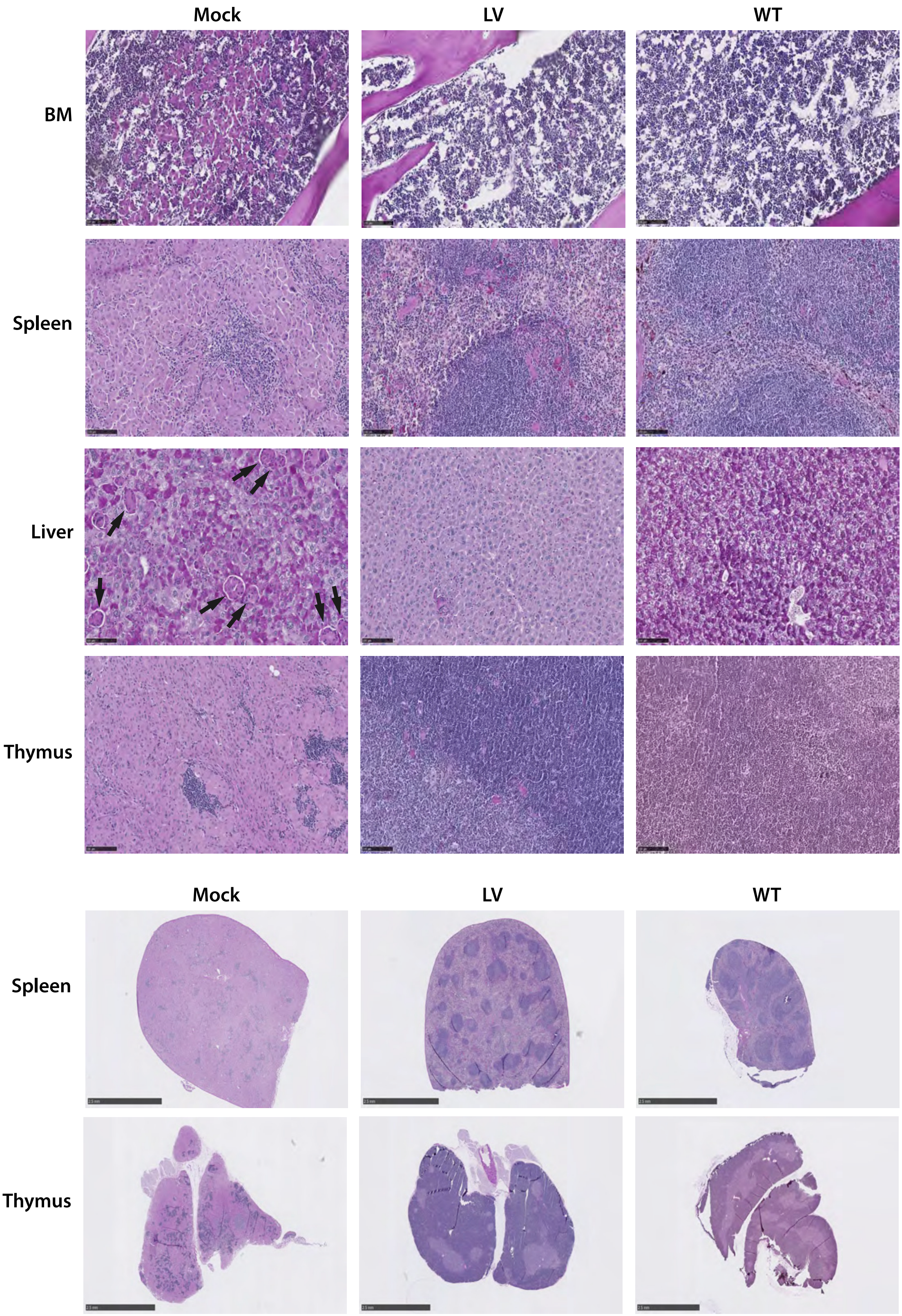

(legend on next page)

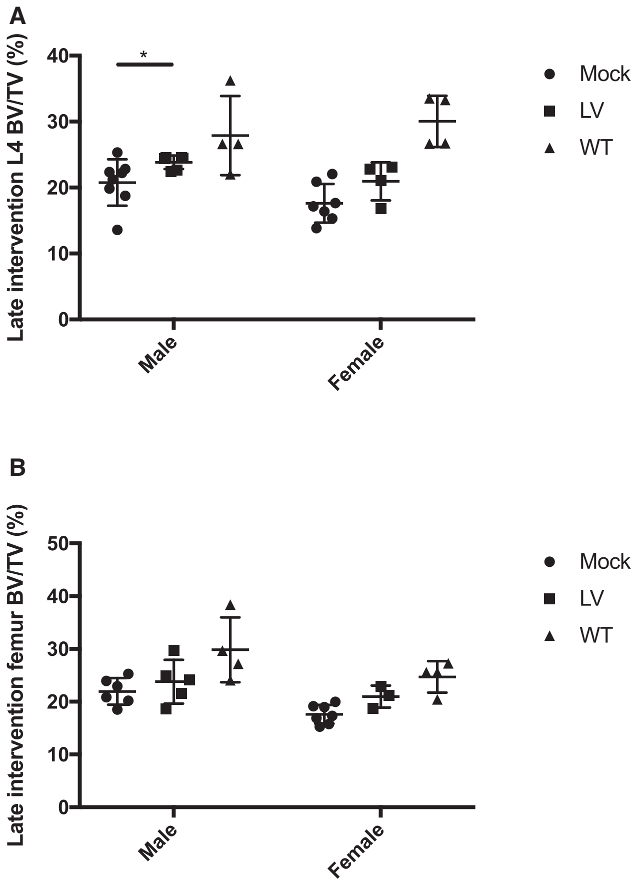

Figure 5. Bone pathology partially corrected by gene therapy in Gaucher mouse model

Micro-CT analysis of spine L4 vertebral body and femur metaphysis area. (A) Bone volume density (BV/TV) of L4 in male and female mice in late intervention study. L4 males; Mock, n = 8; LV, n = 6; WT, n = 4. L4 females; Mock, n = 7; LV, n = 4; WT, n = 4. (B) BV/TV in femur of male and female mice in late intervention study. Femur males; Mock, n = 6; LV, n = 5; WT, n = 4. Femur females; Mock, n = 7; LV, n = 3; WT, n = 4. Additional micro-CT information is in the Supplemental Information (Figures S5 and S6). Mann-Whitney U-test *p ≤ 0.05; **p ≤ 0.01; ***p ≤ 0.001; ****p ≤ 0.0001. Error bars represent mean ± SD.

femur samples from LV-transplanted mice compared to mock (Figure 5B; Figure S6Ai).

In summary, transplantation of LV-transduced cells to GD1 animals resulted in tendencies of increased bone mass and bone density when compared to the mock-transplanted GD1 mice, suggesting a potential for efficacy regarding bone parameters in gene therapy treated animals.

The EFS.GBA SIN lentiviral vector exhibits an oligoclonal integration pattern and a typical lentiviral integration profile in GD1 mice

VCN analyses were performed on BM samples and revealed an average VCN of 0.45 for samples in the early intervention study and 0.43 in the late intervention study (Figures 2E and 3E). Insertion site analysis was then conducted on genomic DNA from nine of the BM samples (from both early and late intervention cohorts) transplanted with LV vector. Integration sites were analyzed for their diversity/polyclonality, distance to transcriptional start sites (TSSs) and other genomic features, clonal imbalance (prominent clones contributing to the overall sequencing pool), proximity to known proto-oncogenes, and the number of shared integrations between different recipients.

Overall, the number of retrieved integration sites in the GD1 mouse model was low, with a mean of 21 ± 16 integrations per sample. Insertions followed a typical lentiviral insertion site pattern with classical preferences for integrating inside the body of genes (Figure 6A). Prominent clones contributing to more than 20% of the overall sequencing pool are detailed in Figure 6B. The relatively high abundance of some clones is due to the low number of insertions observed *in vivo* per mouse resulting in an oligoclonal insertion pattern as compared to the *in vitro* product. Integrations near *Lrrc4c* were found in three of the analyzed samples, as well as in an *in vitro* control sample. The only other overlap between animals was found close to gene *Ibtk*, for which the contribution was below 1% in either case (Table S1). Importantly, no *in vivo* selection was observed for proto-oncogenes whose human orthologs have an association with insertional mutagenesis events (*Lmo2*, *Ikzf1*, *Ccnd2*, *Hmga2*, or *Mecom*). As expected, for animals receiving mock-transduced cells, no valid integration sites were found.

Taken together, the vector displayed an oligoclonal distribution pattern with no sign of vector-induced clonal dominance and a typical lentiviral integration profile.

DISCUSSION

Though effective in alleviating disease symptoms in large cohorts of patients, conventional treatment has the major drawback of being non-curative and expensive, resulting in very significant health care costs over the lifetime of the patient. Gene therapy, entailing *ex vivo* gene augmentation to autologous hematopoietic cells by

Figure 4. Gene therapy diminishes Gaucher cell infiltration and preserves normal tissue architecture

Periodic acid Schiff staining of BM, spleen, liver, and thymus samples from Mock, LV, and WT mice. Mock samples, i.e., untreated mice, present with macrophages engorged with glucocerebroside, which are visualized as multinucleated, light pink cells under the microscope. Clusters of infiltrating Gaucher cells are visible in BM, spleen, and thymus mock samples. Black arrows in the mock liver sample identifies individually scattered Gaucher cells. Panel shows representative samples of female mice in the early intervention study. Representative histology figures from male mice in the early intervention study and from female and male mice of the late intervention study can be found in the Supplemental Information (Figures S2–S4). Upper panel of BM, spleen, liver, and thymus samples; black bars in lower left side represents scale bars of 100 μm. Lower panel of spleen and thymus tissue architecture; black bars in lower left side represents scale bars of 1 mm.

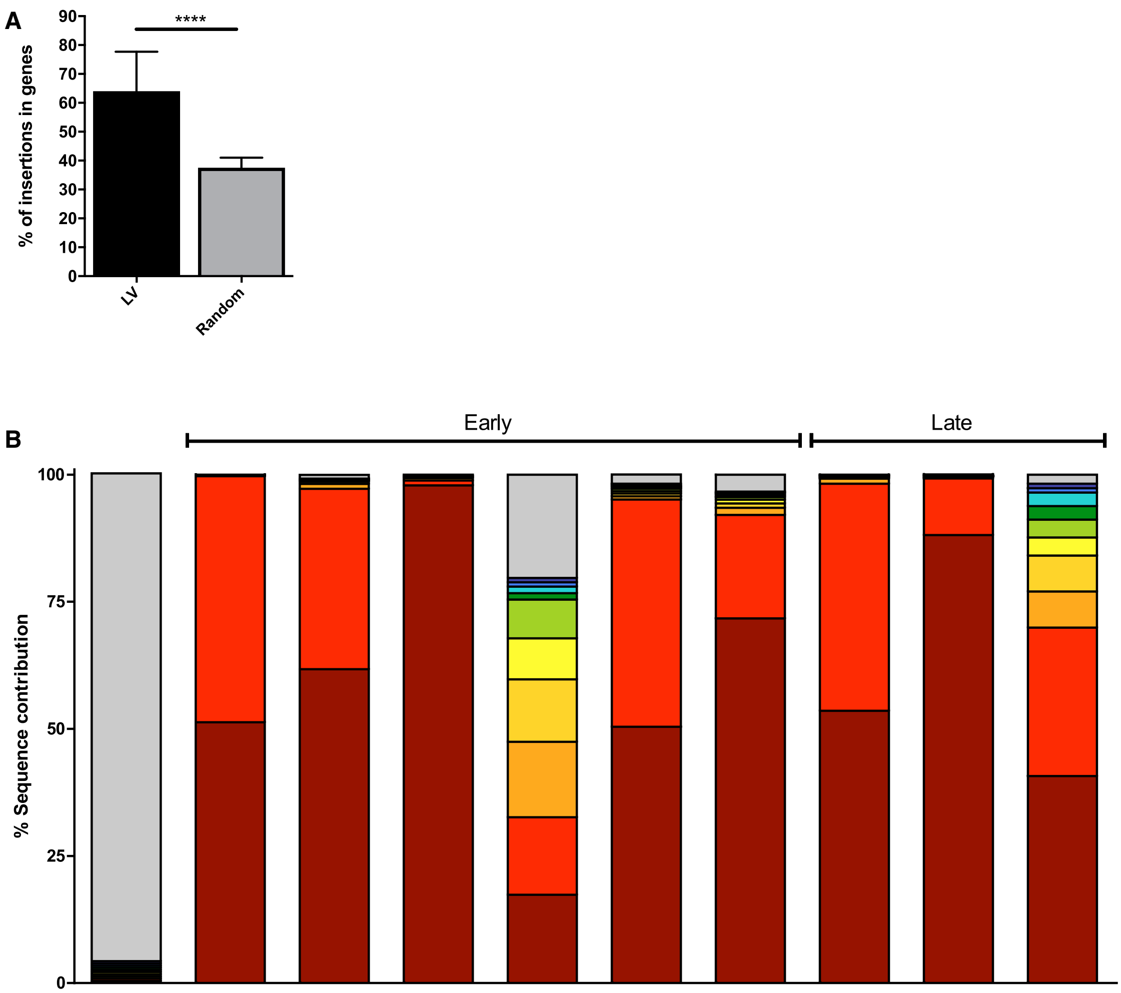

	In vitro	0410	0432	0434	0400	0494	0493	059	086	0242
1	Tmem135~*	Cpsf2~	Enah~*	Trappc8*	Nbas*	Tfpt*	Lin52*	Pde4d~*	1700018B24Rik	Ppargc1a~
2	Lrrc4c	Pola1~*	Ilkap*	Zc3h12b*	Gm20756	Eif3l*	Osbpl1a~*	Nfat5~*	Snhg14*	Etv1
3	Lrrc4c	Scaf8*	Zfp943*	Gtf2a1~*	Ahi1~*	Map3k4*	Ppp1r2-ps9	Tsen2*	Hmgcr	Cdc14a~
4	Lrrc20~*	Pafah1b1*	Arsk*	Gm595~	Gm14664	1190002N15Rik	Srgap2~*	Kif13b*	Smc6~*	9330182L06Rik*
5	Abhd2~	Washc4*	Pet2	Angpt1~	Socs2	Lin9~*	Mapk14*	Ip6k1*	Nfatc3~*	Sec61g*
6	Greb1*	Ibtk*	Lrrc4c	Cenpe*	Mir195b	Scfd2*	Tmem161b*	Gm13582	Brwd3*	Fcna*
7	Asic2*	Clptm1l	Zfp324~		Lingo2~*	Primpol*	Alcam~*	Tbc1d32*	D17Wsu92e*	Tet2*
8	Filip1*	Gsk3b*	Mir99ahg*		Nae1*	Map3k4*	Stx8~*	Celf1*	Cabp5	Sprr2g
9	Abi1*		Pomgnt2*		Aoah~	Arhgap15~*	Tnfaip8~*	Ftmt	Atxn2*	Ibtk*
10	Add3*		Epc2*		4930486L24Rik*	Ifi47~*	Loxl2~*	Adam6b	Lrrc4c	Mcmbp*
UIS	425	8	24	6	55	32	29	10	10	12
**VCN**	**186.52**	**0.94**	**1.11**	**0.61**	**0.16**	**1.43**	**0.73**	**0.44**	**1.58**	**0.52**

Figure 6. Vector analysis reveals oligoclonal integration profile with no detectable insertions into known high-risk proto oncogenes
The following BM samples were chosen for integration analysis by the INSPIIRED platform; #059, 086, 0242, 0400, 0410, 0432, 0434, 0493, and 0494. Two Mock BM samples were used as negative controls (#0402 and 0495), these did not generate any valid integration sites. (A) LV samples were compared to matched random controls (part of the INSPIIRED pipeline) in terms of percentage (%) of insertions inside of genes. Mann-Whitney U-test *$p \leq 0.05$; **$p \leq 0.01$; ***$p \leq 0.001$; ****$p \leq 0.0001$. Error bars represent mean + SD. (B) The ten integrations with the highest sequence contribution in each analyzed sample are depicted. The gene symbol closest to the integration site are shown. According to the INSPIIRED pipeline, an asterisk (*) behind the gene symbol indicates that the integration was within a transcription unit and a tilde (~) that the insertion was within 50 kb of a cancer-related gene.

state-of-the-art lentiviral vectors, has the potential of becoming a future treatment option for Gaucher disease type 1 patients. Hematopoietic stem cell gene therapy (HSCGT) circumvents the need for finding an immune-compatible donor and thereby avoids the risks associated with allo-HSCT such as graft-versus-host disease. Therefore, the serious complications associated with allo-HSCT would be avoided with an autologous transplant. ERT has well-documented long-term safety data, however, the current management by ERT encompasses regular, bi-weekly infusions of recombinant protein, with associated antibody and other immune responses developing over time. It is our belief that a gene therapy approach could prove beneficial for those individuals where conventional treatment is deemed insufficient in managing the full set of symptoms experienced by the patient. As a presumable 'one-time' curative approach, the prospective advantages of HSCGT are clear.

We have investigated the efficacy and safety of a SIN lentiviral vector containing an RNA transcript that, after reverse transcription, results in codon-optimized cDNA that, upon its integration into the human genome, encodes for functional human glucocerebrosidase driven by the EFS promoter, a cellular promoter employed in several clinical trials including X-linked severe combined immunodeficiency (SCID-X1) (ClinicalTrials.gov: NCT01410019, NCT01129544, NCT01306019) and adenosine deaminase severe combined immunodeficiency (ADA-SCID) (ClinicalTrials.gov: NCT01380990, NCT01852071, NCT02999984). From our studies, we conclude that even a low level of gene transfer through the EFS.GBA SIN LV vector (VCN < 0.5) effectively prevents pathophysiological manifestations in the GD1 animal model. Compared to mock-treated mice, treated animals exhibited substantially lower levels of substrate in all assessed organs. A concomitant reduction of macrophages engorged with unprocessed sphingolipids (Gaucher cells) was observed, which to a large extent preserved normal organ architecture in spleen and thymus. Furthermore, compared to untreated mice, treated animals had significant reductions in spleen and liver mass and older GD1 mice also exhibited a correction in red blood cell parameters. Even though the gene therapy treatment proved most efficacious in a setting with relatively low levels of accumulated substrate (as in the early intervention approach), lentiviral gene therapy had impressive therapeutic effect on physical signs of manifest Gaucher disease considering the relatively low level of transduced cells (on average 0.46 copies per cell across tissues, late intervention). These findings are consistent with previous studies conducted in our laboratory showing that only a modest increase in GCase activity, provided by a low percentage of macrophages/myeloid cells expressing functional enzyme, was sufficient to reverse pathophysiological signs in GD1 mice.[21,27] Based on the results presented in this article along with our previously published data, we envision that even a small number of engrafted gene-corrected HSPCs will potentially have highly beneficial effects in patients. Importantly, we have not observed any selective advantage for gene-corrected cells in our Gaucher disease animal model. This is consistent with findings from the initial clinical trial including three GD type 1 patients. These patients, devoid of any pretransplant myeloablation, were infused with gene-corrected autologous $CD34^+$ which yielded only transient expression of detectable transduced cells.[28] This early clinical trial highlights the importance of including a myeloablative conditioning regime in a future transplant protocol for GD1 to ensure long-term engraftment of gene-corrected, repopulating cells. Previous findings by our group also describes the use of busulfan with a WT cell engraftment in the range of 1%–10% as able to confer beneficial therapeutic outcome in the GD1 mouse model.[27]

Clinical studies using dual-energy quantitative CT and dual-energy X-ray absorptiometry to investigate skeletal involvement in GD1 patients have revealed that mean bone density at multiple skeletal sites are significantly lower than expected for age and sex.[29,30] Data from the ICGG Gaucher Registry have shown that a low bone mineral mass (BMD) of the lumbar spine (L1–L4) is associated with a high risk of fracture of the spine or femur in GD1 patients.[31] In general, bone mass and density changes were observed in the LV-transduced GD1 mice in this study as evidenced by increases in related micro-CT parameters (BV/TV, vBMT, BV, and BMC) when compared to mock-transplanted controls. As the GD1 mouse strain has not been characterized in terms of bone disease-related changes in this, nor in any previous studies, caution is advised when interpreting the micro-CT results in this study in relation to bone pathology observed in Gaucher disease patients. Additionally, proper comparison on bone parameters required groups be divided based on sex, i.e., the number of animals in each group were limited. In the early intervention cohorts, effects were generally greater in males than females, and were mainly related to increased bone size. The age difference between mice in the early and late intervention study explains in part why bone size is mostly affected in the early intervention males as bone growth is not fully completed in 11- 16-week-old mice, especially in males.[32] Despite the above-mentioned reservations, the observed tendencies of increased bone mass and density in LV-transplanted GD1 mice are of interest since conventional treatment regimens offer little to no effect on the debilitating bone pathology experienced by many patients.

We detected only a low number of lentiviral vector integrations per mouse in our study. The observed oligoclonality might be due to low VCN per cell. Overall, the insertions followed a typical lentiviral preference to integrate inside of transcriptional units. No integrations in or close to known high-risk proto-oncogenes (*Lmo2*, *Ikzf1*, *Ccnd2*, *Hmga2*, or *Mecom*) were found.

In summary, we conclude that the EFS.GBA lentiviral vector is efficacious in reducing substrate levels and preventing (as well as reversing) pathophysiological signs that arise in the Gaucher disease type 1 animal model, along with exhibiting a typical lentiviral integration profile. We propose this vector be used in an investigational clinical study for treating Gaucher disease type 1 in conjunction with a myeloablative conditioning regimen.

MATERIALS AND METHODS

GD1 mice

The generation of the GD1 mouse model has been previously described.[22] Screening of mice for experiments after polyinosinic-polycytidylic acid (pIpC) (Sigma-Aldrich)-induced exon deletion was

done by PCR using platinum Taq polymerase (Invitrogen). The following primers were used to confirm the presence of the Cre gene: (5′-AATGCTTCTGTCCGTTTGCCGGTC-3′; 5′-GATCCGTCGCATGACCAGTGAAAC-3′). These primers were used to screen for GBA1 null animals; (5′-TAGAGTCCCTCCAGCTTCCCAG-3′; 5′-GTACGTTCATGGCATTGCTGTTCACT-3′; 5′-ATTCCAGCTGTCCCTCGTCTCC-3′). Mice were maintained in individually ventilated cages with *ad libitum* food and water in the animal facility at Lund University Biomedical Center. GD1 donor and recipients were from the same litters and of similar age. Experimental cohorts included both female and male mice. Breeding and experimental procedures were approved by the Committee for Animal Ethics in Malmö/Lund, Sweden.

Vector

The vector used in this study is a third-generation SIN lentiviral vector containing a ribonucleic acid (RNA) transcript that, after reverse transcription, results in codon-optimized cDNA that, upon its integration into the human genome, encodes for functional human glucocerebrosidase under the control of an EFS promoter. Vesicular stomatitis virus G protein (VSV-G)-pseudotyped vector was produced at a Good Manufacturing Practice (GMP) certified facility (Lentigen Technology, Gaithersburg, MD, USA). The vector batch used in this study was of research-grade quality (i.e., non-GMP/preclinical).

BM cell purification, transduction, and transplantation

Bone marrow from donor GD1 animals was harvested, crushed in 2% fetal bovine serum (FBS, GE Healthcare Life Sciences) in phosphate buffered saline (PBS; GE Healthcare Life Sciences) and filtered. Bone marrow cells were then lineage depleted of mature blood cell subsets using MACS cell separation (Miltenyi Biotec) according to manufacturer's instructions. Lin^- cells were kept in StemSpan Serum-Free Expansion Medium (SFEM, StemCell Technologies) supplemented with 1% penicillin-streptomycin (P/S, GE Healthcare Life Sciences) and the following cytokine combination; 100 ng/mL mSCF (Peprotech), 10 ng/mL mIL3 (Peprotech), 50 ng/mL hTPO (Peprotech), and 10 ng/mL hIL6 (Peprotech). Cells were transduced overnight at a multiplicity of infection (MOI) of 20 in wells coated with RetroNectin (Takara Clontech). Cells were cultured overnight in a humidified incubator at 37°C 5% CO_2. The following day, the vector-containing media was removed, and cells resuspended in freshly prepared media. Mock cells were not transduced but otherwise prepared and cultured in the same manner as transduced cells. Recipient mice were lethally irradiated (900 cGy) 4 h before tail vein infusion of 200,000 viable Lin^- cells per mouse. Donor and recipient mice were of similar age and cohorts contained a mix of male and female mice. A small aliquot of both mock and transduced cells was saved and evaluated in terms of GCase activity along with transduction efficiency and VCN insertion per diploid genome 48 h after transduction. To estimate transduction efficiency, cells were seeded in semisolid media containing cytokines (MethoCult GF M3434, StemCell Technologies). After 10–12 days of culture, individual colonies were picked and used in PCR reactions for detection of WPRE element (backbone component of the lentiviral vector) to assess the percentage of vector transfer to individual cells.

Tissue and cell preparations at harvest

Blood samples were collected in EDTA-coated Microvette collection tubes (Sarstedt). Hematology analyses were performed using a Sysmex XE-5000 automated cell counter at room temperature. Mice were weighed prior to sacrifice and femur, tibiae, iliac, humerus, spine, liver, spleen, and thymus collected at necropsy. Spleens and livers were weighed and tissues for VCN analysis snap frozen in liquid nitrogen immediately after weighing. BM cells for VCN and insertional site analysis were kept in PBS (2% FCS) and later crushed, filtered, counted, and frozen. For histopathology, tissue samples were immediately submerged in 4% paraformaldehyde after collection.

VCN and insertional site analysis

Mean VCN were determined by quantitative real-time PCR. A primer-probe set designed to detect the lentiviral backbone (modified WPRE) and a housekeeping gene, respectively, were used to quantify the amount of vector insertions per cell.[33] The VCN in BM samples was either determined by quantitative real-time PCR in a StepOnePlus (Thermo Fisher Scientific) or Droplet Digital PCR (ddPCR) using a QX200 system (Bio-Rad). Quantitative analysis of integration site distributions was performed using the INtegration Site PIpeline for paIRED-ends reads (INSPIIRED)[34,35] platform on whole BM genomic DNA (further technical details can be provided upon request).

Glucocerebroside (GlcCer) and glucosylsphingosine (GlcSph) quantification

GlcCer and GlcSph content was evaluated in BM, spleen, and liver samples. Briefly, GlcCer and GlcSph were extracted by a modification of the Bligh and Dyer method using acidic buffer (100 mM ammonium formate buffer pH 3.1). Prior to extraction, 20 μL of the internal standard C17-dh-Ceramide (20 μM) and 20 μL 13C5-GlcSph (0.1 μM) were added to the homogenate. Briefly, lipids were extracted by adding methanol, chloroform, and ammonium formate buffer (1:1:0.9; v/v/v), which resulted in 2 phases. The upper phase was dried under N2 stream and further extracted with water/butanol (1:1; v/v) before being applied to the UPLC-MS. The lower phase was transferred to a Pyrex tube and deacylated in a microwave for 1 h with 500 μL of methanolic NaOH (0.1 M). Deacylated lipids were extracted with water/butanol (1:1; v/v) and applied to the UPLC-MS. Lipids were analyzed by reverse-phase liquid chromatography using a Waters UPLC-Xevo-TQS micro and a BEH C18 column, 2.1 × 50 mm with 1.7 μm particle size (Waters, USA). Data were processed with MassLynx 4.1 software (Waters Corporation, USA). The method has been described previously.[36]

Histopathology analysis of Gaucher cell infiltration in hematopoietic tissues

Fixed left tibia (decalcified), spleen, liver, and thymus tissue were paraffin embedded and sectioned in 5 μm sections, mounted on glass

slides, and stained with hematoxylin and eosin and periodic acid Schiff staining for microscopic examination.

Micro-CT analysis of bone

The left or right femur and L4 vertebral body (or L5 in case L4 was unavailable) were subjected to bone densitometry evaluation using a high-resolution micro-CT system. For the vertebral body and for the femur distal metaphysis, one volume of interest (VOI) was defined, contoured, and analyzed. Individual data were tabulated to include 3D morphometric parameters.

SUPPLEMENTAL INFORMATION

Supplemental Information can be found online at https://doi.org/10.1016/j.omtm.2020.11.018.

ACKNOWLEDGMENTS

The authors thank personnel at Charles River Laboratories (Wilmington, NC, USA) for histopathological evaluation of BM, spleen, liver, and thymus samples and Charles River Laboratories (Montreal, QC, Canada) for micro-CT analysis of bone. Additionally, we thank Lovelace Biomedical (Albuquerque, NM, USA) for VCN analysis in blood, liver, and spleen tissues.

AUTHOR CONTRIBUTIONS

S.K. and C.M. conceptualized the project. M.D., E.M.K.S., and S.K. designed experiments and wrote the paper. M.D., E.M.K.S., S.W., M.J.F., and M.R. performed experiments and analyzed data. A.S. and C.M. provided vector. J.M.F.G.A., A.S., M.R., A.G., C.H., R.P., and D.P. provided expertise and feedback.

DECLARATION OF INTERESTS

This work was fully funded by AVROBIO (Cambridge, MA, USA). C.M., A.G., C.H., R.P., and D.P. are employees at AVROBIO. A.S., S.K., and M.D. are employed consultants for AVROBIO.

REFERENCES

1. Brady, R.O., Kanfer, J.N., and Shapiro, D. (1965). Metabolism of Glucocerebrosides. Ii. Evidence of an Enzymatic Deficiency in Gaucher's Disease. Biochem. Biophys. Res. Commun. *18*, 221–225.
2. Grabowski, G.A. (2008). Phenotype, diagnosis, and treatment of Gaucher's disease. Lancet *372*, 1263–1271.
3. Stirnemann, J., Belmatoug, N., Camou, F., Serratrice, C., Froissart, R., Caillaud, C., Levade, T., Astudillo, L., Serratrice, J., Brassier, A., et al. (2017). A Review of Gaucher Disease Pathophysiology, Clinical Presentation and Treatments. Int. J. Mol. Sci. *18*, 441.
4. Thomas, A.S., Mehta, A., and Hughes, D.A. (2014). Gaucher disease: haematological presentations and complications. Br. J. Haematol. *165*, 427–440.
5. Hollak, C.E., and Weinreb, N.J. (2015). The attenuated/late onset lysosomal storage disorders: Therapeutic goals and indications for enzyme replacement treatment in Gaucher and Fabry disease. Best Pract. Res. Clin. Endocrinol. Metab. *29*, 205–218.
6. Hill, S.C., Reinig, J.W., Barranger, J.A., Fink, J., and Shawker, T.H. (1986). Gaucher disease: sonographic appearance of the spleen. Radiology *160*, 631–634.
7. Stirnemann, J., Vigan, M., Hamroun, D., Heraoui, D., Rossi-Semerano, L., Berger, M.G., Rose, C., Camou, F., de Roux-Serratrice, C., Grosbois, B., et al. (2012). The French Gaucher's disease registry: clinical characteristics, complications and treatment of 562 patients. Orphanet J. Rare Dis. *7*, 77.
8. Rosenbaum, H. (2014). Hemorrhagic aspects of Gaucher disease. Rambam Maimonides Med. J. *5*, e0039.
9. Wenstrup, R.J., Roca-Espiau, M., Weinreb, N.J., and Bembi, B. (2002). Skeletal aspects of Gaucher disease: a review. Br. J. Radiol. *75* (*Suppl 1*), A2–A12.
10. Marcucci, G., Zimran, A., Bembi, B., Kanis, J., Reginster, J.Y., Rizzoli, R., Cooper, C., and Brandi, M.L. (2014). Gaucher disease and bone manifestations. Calcif. Tissue Int. *95*, 477–494.
11. Riboldi, G.M., and Di Fonzo, A.B. (2019). *GBA*, Gaucher Disease, and Parkinson's Disease: From Genetic to Clinic to New Therapeutic Approaches. Cells *8*, 364.
12. Weinreb, N.J., Charrow, J., Andersson, H.C., Kaplan, P., Kolodny, E.H., Mistry, P., Pastores, G., Rosenbloom, B.E., Scott, C.R., Wappner, R.S., and Zimran, A. (2002). Effectiveness of enzyme replacement therapy in 1028 patients with type 1 Gaucher disease after 2 to 5 years of treatment: a report from the Gaucher Registry. Am. J. Med. *113*, 112–119.
13. Charrow, J., Dulisse, B., Grabowski, G.A., and Weinreb, N.J. (2007). The effect of enzyme replacement therapy on bone crisis and bone pain in patients with type 1 Gaucher disease. Clin. Genet. *71*, 205–211.
14. Mohty, B., and Mohty, M. (2011). Long-term complications and side effects after allogeneic hematopoietic stem cell transplantation: an update. Blood Cancer J. *1*, e16.
15. Cavazzana, M., Bushman, F.D., Miccio, A., André-Schmutz, I., and Six, E. (2019). Gene therapy targeting haematopoietic stem cells for inherited diseases: progress and challenges. Nat. Rev. Drug Discov. *18*, 447–462.
16. Marquez Loza, L.I., Yuen, E.C., and McCray, P.B., Jr. (2019). Lentiviral Vectors for the Treatment and Prevention of Cystic Fibrosis Lung Disease. Genes (Basel) *10*, 218.
17. Cartier, N., Hacein-Bey-Abina, S., Bartholomae, C.C., Veres, G., Schmidt, M., Kutschera, I., Vidaud, M., Abel, U., Dal-Cortivo, L., Caccavelli, L., et al. (2009). Hematopoietic stem cell gene therapy with a lentiviral vector in X-linked adrenoleukodystrophy. Science *326*, 818–823.
18. Eichler, F., Duncan, C., Musolino, P.L., Orchard, P.J., De Oliveira, S., Thrasher, A.J., Armant, M., Dansereau, C., Lund, T.C., Miller, W.P., et al. (2017). Hematopoietic Stem-Cell Gene Therapy for Cerebral Adrenoleukodystrophy. N. Engl. J. Med. *377*, 1630–1638.
19. Biffi, A., Montini, E., Lorioli, L., Cesani, M., Fumagalli, F., Plati, T., Baldoli, C., Martino, S., Calabria, A., Canale, S., et al. (2013). Lentiviral hematopoietic stem cell gene therapy benefits metachromatic leukodystrophy. Science *341*, 1233158.
20. Sessa, M., Lorioli, L., Fumagalli, F., Acquati, S., Redaelli, D., Baldoli, C., Canale, S., Lopez, I.D., Morena, F., Calabria, A., et al. (2016). Lentiviral haemopoietic stem-cell gene therapy in early-onset metachromatic leukodystrophy: an ad-hoc analysis of a non-randomised, open-label, phase 1/2 trial. Lancet *388*, 476–487.
21. Dahl, M., Doyle, A., Olsson, K., Månsson, J.E., Marques, A.R.A., Mirzaian, M., Aerts, J.M., Ehinger, M., Rothe, M., Modlich, U., et al. (2015). Lentiviral gene therapy using cellular promoters cures type 1 Gaucher disease in mice. Mol. Ther. *23*, 835–844.
22. Enquist, I.B., Nilsson, E., Ooka, A., Månsson, J.E., Olsson, K., Ehinger, M., Brady, R.O., Richter, J., and Karlsson, S. (2006). Effective cell and gene therapy in a murine model of Gaucher disease. Proc. Natl. Acad. Sci. USA *103*, 13819–13824.
23. Beighton, P., Goldblatt, J., and Sacks, S. (1982). Bone involvement in Gaucher disease. Prog. Clin. Biol. Res. *95*, 107–129.
24. Green, D.E., Adler, B.J., Chan, M.E., Lennon, J.J., Acerbo, A.S., Miller, L.M., and Rubin, C.T. (2013). Altered composition of bone as triggered by irradiation facilitates the rapid erosion of the matrix by both cellular and physicochemical processes. PLoS ONE *8*, e64952.
25. Hamilton, S.A., Pecaut, M.J., Gridley, D.S., Travis, N.D., Bandstra, E.R., Willey, J.S., Nelson, G.A., and Bateman, T.A. (2006). A murine model for bone loss from therapeutic and space-relevant sources of radiation. J. Appl. Physiol. *101* (1985), 789–793.
26. Kondo, H., Searby, N.D., Mojarrab, R., Phillips, J., Alwood, J., Yumoto, K., Almeida, E.A., Limoli, C.L., and Globus, R.K. (2009). Total-body irradiation of postpubertal mice with (137)Cs acutely compromises the microarchitecture of cancellous bone and increases osteoclasts. Radiat. Res. *171*, 283–289.
27. Enquist, I.B., Nilsson, E., Månsson, J.E., Ehinger, M., Richter, J., and Karlsson, S. (2009). Successful low-risk hematopoietic cell therapy in a mouse model of type 1 Gaucher disease. Stem Cells *27*, 744–752.

28. Dunbar, C.E., Kohn, D.B., Schiffmann, R., Barton, N.W., Nolta, J.A., Esplin, J.A., Pensiero, M., Long, Z., Lockey, C., Emmons, R.V., et al. (1998). Retroviral transfer of the glucocerebrosidase gene into CD34+ cells from patients with Gaucher disease: in vivo detection of transduced cells without myeloablation. Hum. Gene Ther. *9*, 2629–2640.

29. Rosenthal, D.I., Barton, N.W., McKusick, K.A., Rosen, B.R., Hill, S.C., Castronovo, F.P., Brady, R.O., Doppelt, S.H., and Mankin, H.J. (1992). Quantitative imaging of Gaucher disease. Radiology *185*, 841–845.

30. Pastores, G.M., Wallenstein, S., Desnick, R.J., and Luckey, M.M. (1996). Bone density in Type 1 Gaucher disease. J. Bone Miner. Res. *11*, 1801–1807.

31. Khan, A., Hangartner, T., Weinreb, N.J., Taylor, J.S., and Mistry, P.K. (2012). Risk factors for fractures and avascular osteonecrosis in type 1 Gaucher disease: a study from the International Collaborative Gaucher Group (ICGG) Gaucher Registry. J. Bone Miner. Res. *27*, 1839–1848.

32. Zoetis, T., Tassinari, M.S., Bagi, C., Walthall, K., and Hurtt, M.E. (2003). Species comparison of postnatal bone growth and development. Birth Defects Res. B Dev. Reprod. Toxicol. *68*, 86–110.

33. Rothe, M., Rittelmeyer, I., Iken, M., Rüdrich, U., Schambach, A., Glage, S., Manns, M.P., Baum, C., Bock, M., Ott, M., and Modlich, U. (2012). Epidermal growth factor improves lentivirus vector gene transfer into primary mouse hepatocytes. Gene Ther. *19*, 425–434.

34. Sherman, E., Nobles, C., Berry, C.C., Six, E., Wu, Y., Dryga, A., Malani, N., Male, F., Reddy, S., Bailey, A., et al. (2016). INSPIIRED: A Pipeline for Quantitative Analysis of Sites of New DNA Integration in Cellular Genomes. Mol. Ther. Methods Clin. Dev. *4*, 39–49.

35. Berry, C.C., Nobles, C., Six, E., Wu, Y., Malani, N., Sherman, E., Dryga, A., Everett, J.K., Male, F., Bailey, A., et al. (2016). INSPIIRED: Quantification and Visualization Tools for Analyzing Integration Site Distributions. Mol. Ther. Methods Clin. Dev. *4*, 17–26.

36. Mirzaian, M., Wisse, P., Ferraz, M.J., Marques, A.R.A., Gaspar, P., Oussoren, S.V., Kytidou, K., Codée, J.D.C., van der Marel, G., Overkleeft, H.S., and Aerts, J.M. (2017). Simultaneous quantitation of sphingoid bases by UPLC-ESI-MS/MS with identical ^{13}C-encoded internal standards. Clin. Chim. Acta *466*, 178–184.

OMTM, Volume *20*

Supplemental Information

Correction of pathology in mice displaying Gaucher disease type 1 by a clinically-applicable lentiviral vector

Maria Dahl, Emma M.K. Smith, Sarah Warsi, Michael Rothe, Maria J. Ferraz, Johannes M.F.G. Aerts, Azadeh Golipour, Claudia Harper, Richard Pfeifer, Daniella Pizzurro, Axel Schambach, Chris Mason, and Stefan Karlsson

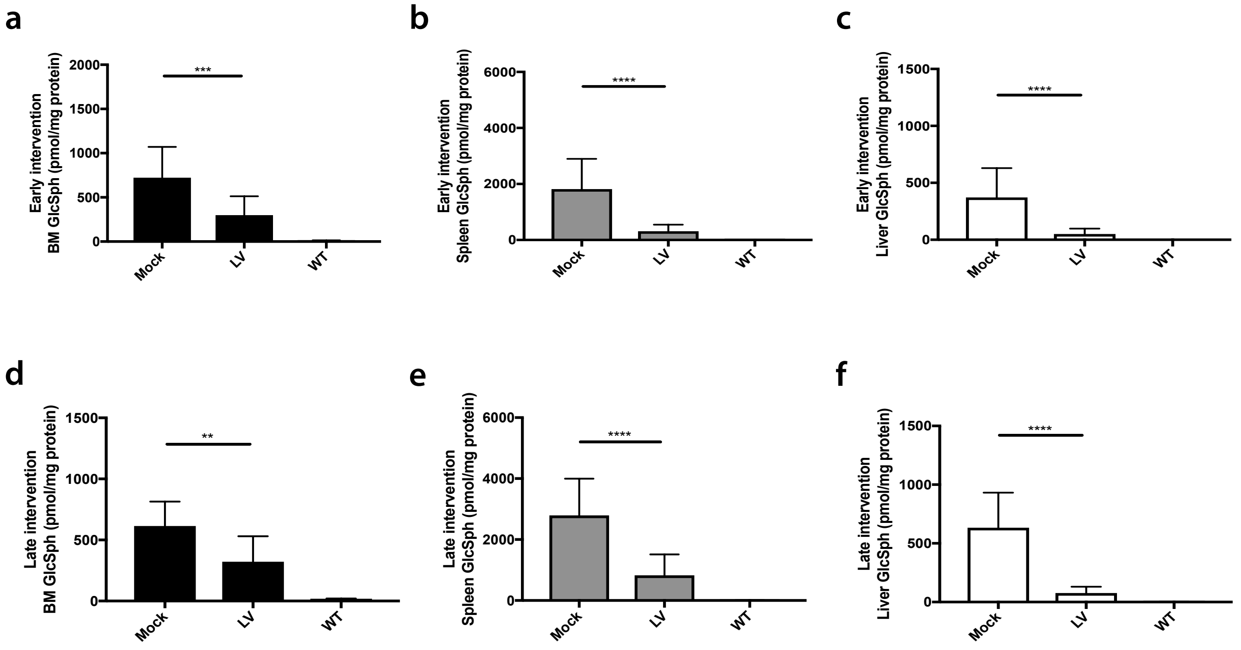

Fig S1) Drastically lowered Glucosylsphingosine accumulation in hematopoietic tissues after gene therapy.

Glucosylsphingosine (GlcSph) content was analyzed in bone marrow (BM), spleen and liver of mice from the Early and Late intervention study. Early intervention study (**a-c**); Mock, *n=13*; LV, *n=13*; WT, *n=8*. Late intervention study (**d-f**); Mock, *n=15*; LV, *n=10*; WT, *n=8*). Mann-Whitney *U*-test; *p = ≤ 0.05; **p = ≤ 0.01; ***p = ≤ 0.001; **** = p = ≤ 0.0001. Error bars represent mean + SD.

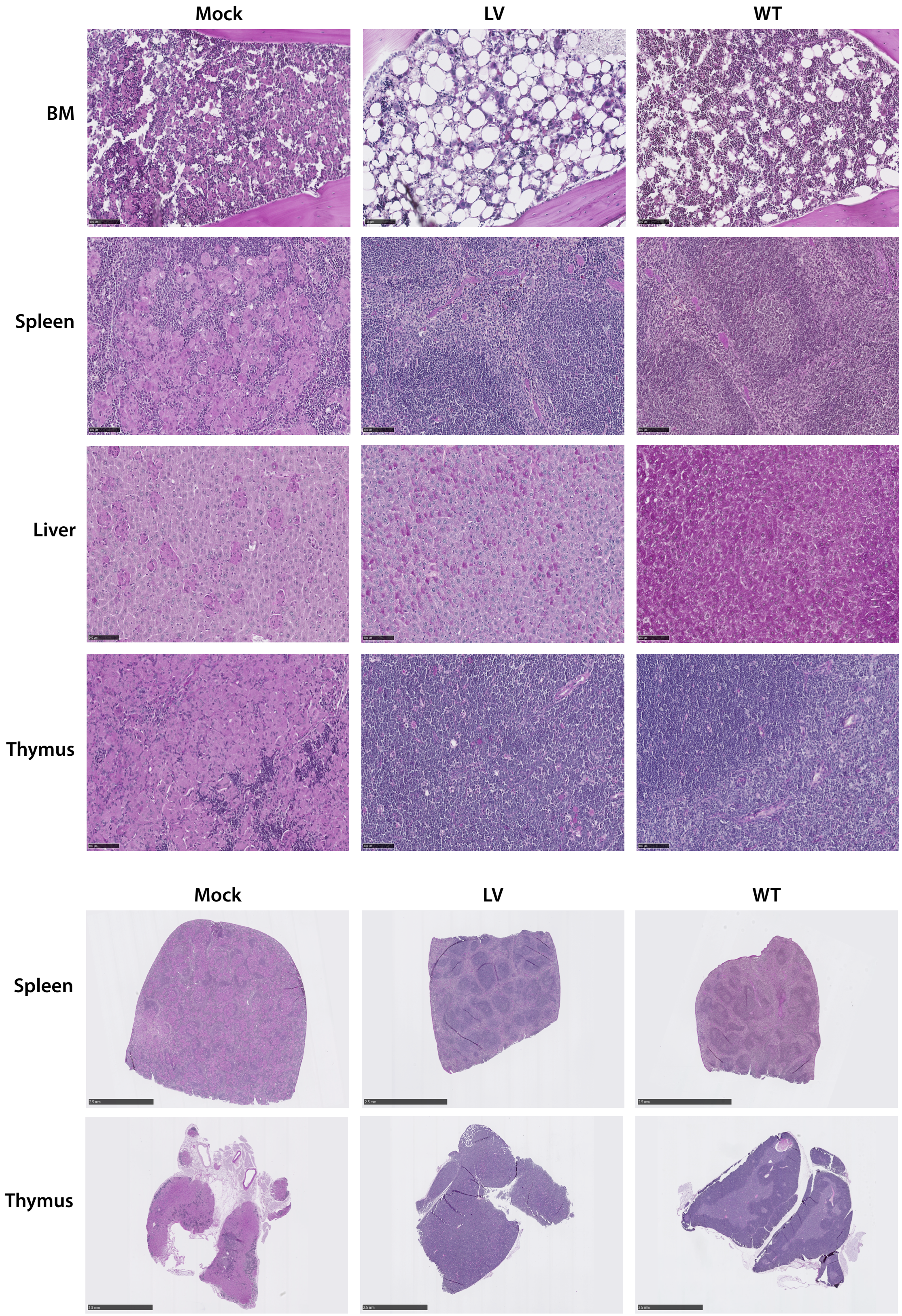

Fig S2) Representative samples of Gaucher cell infiltration in males of Early intervention study.

Periodic Acid Schiff staining of BM, spleen, liver and thymus samples from representative Mock, LV and WT male mice in the Early intervention study. Upper panel of BM, spleen, liver and thymus samples; black bars in lower left side represents scale bars of 100 µm. Lower panel of spleen and thymus tissue architecture; black bars in lower left side represents scale bars of 2.5 mm.

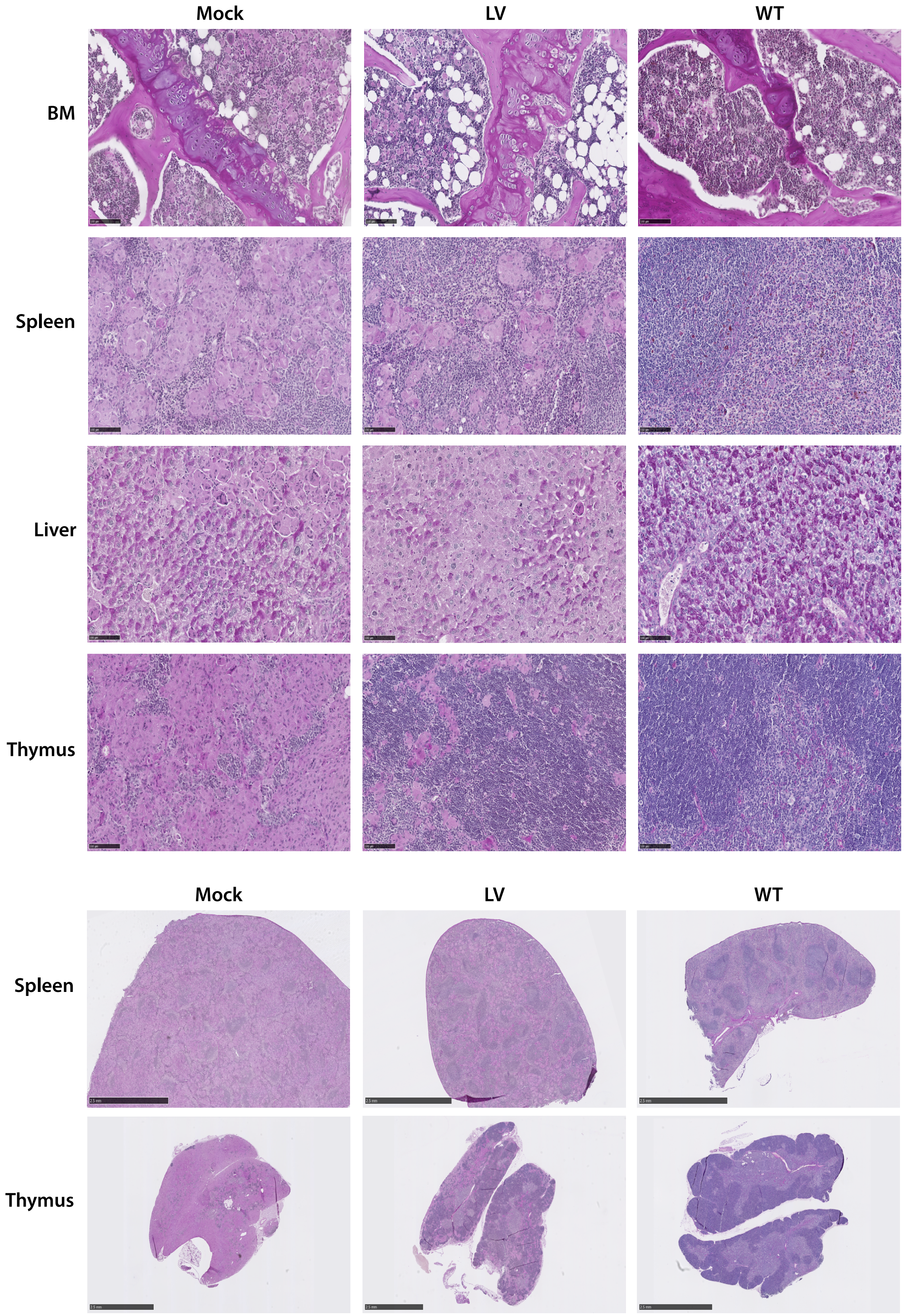

Fig S3) Representative samples of Gaucher cell infiltration in females of Late intervention study.

Periodic Acid Schiff staining of BM, spleen, liver and thymus samples from representative Mock, LV and WT female mice in the Late intervention study. Upper panel of BM, spleen, liver and thymus samples; black bars in lower left side represents scale bars of 100 µm. Lower panel of spleen and thymus tissue architecture; black bars in lower left side represents scale bars of 2.5 mm.

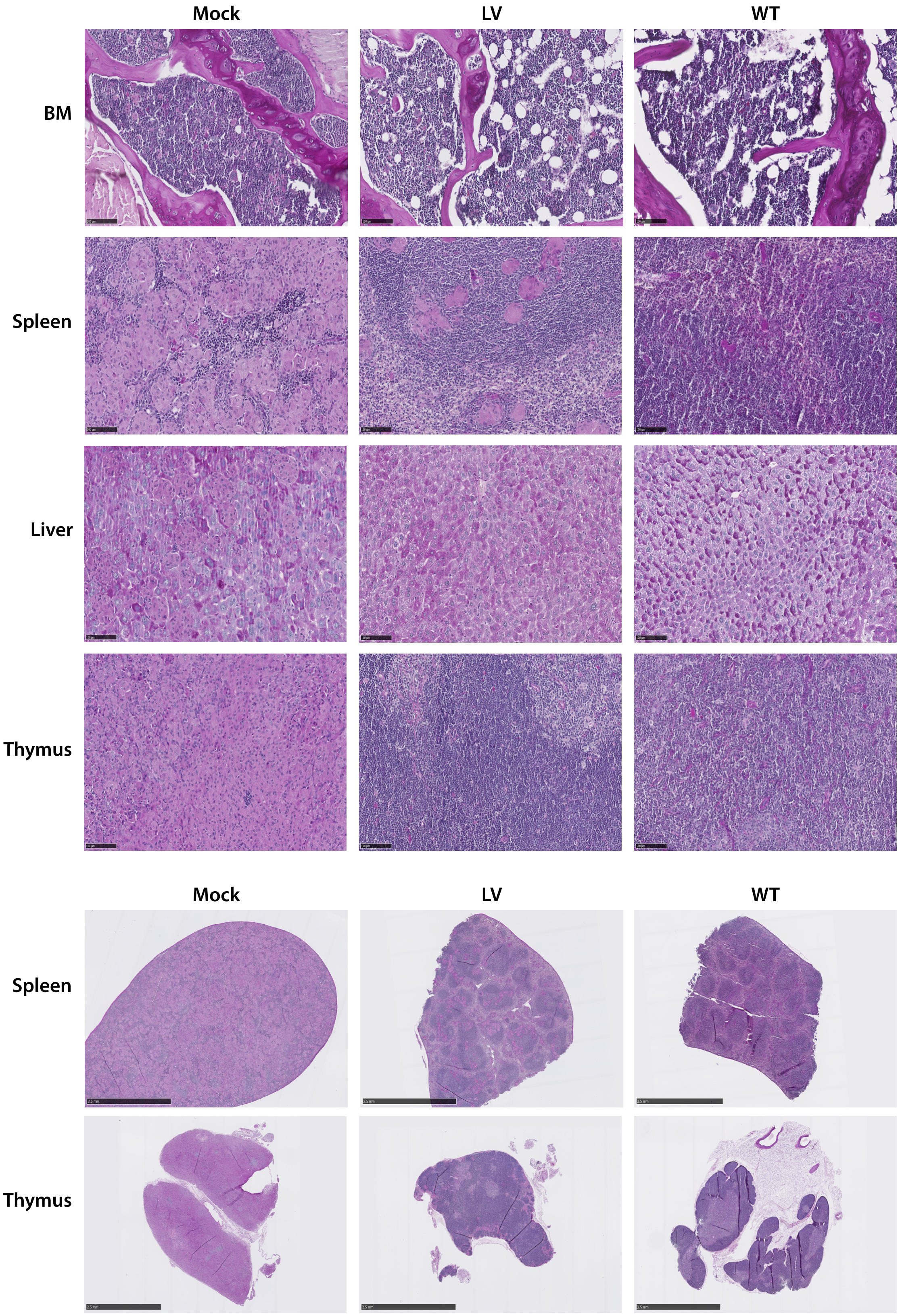

Fig S4) Representative samples of Gaucher cell infiltration in males of Late intervention study.

Periodic Acid Schiff staining of BM, spleen, liver and thymus samples from representative Mock, LV and WT male mice in the Late intervention study. Upper panel of BM, spleen, liver and thymus samples; black bars in lower left side represents scale bars of 100 μm. Lower panel of spleen and thymus tissue architecture; black bars in lower left side represents scale bars of 2.5 mm.

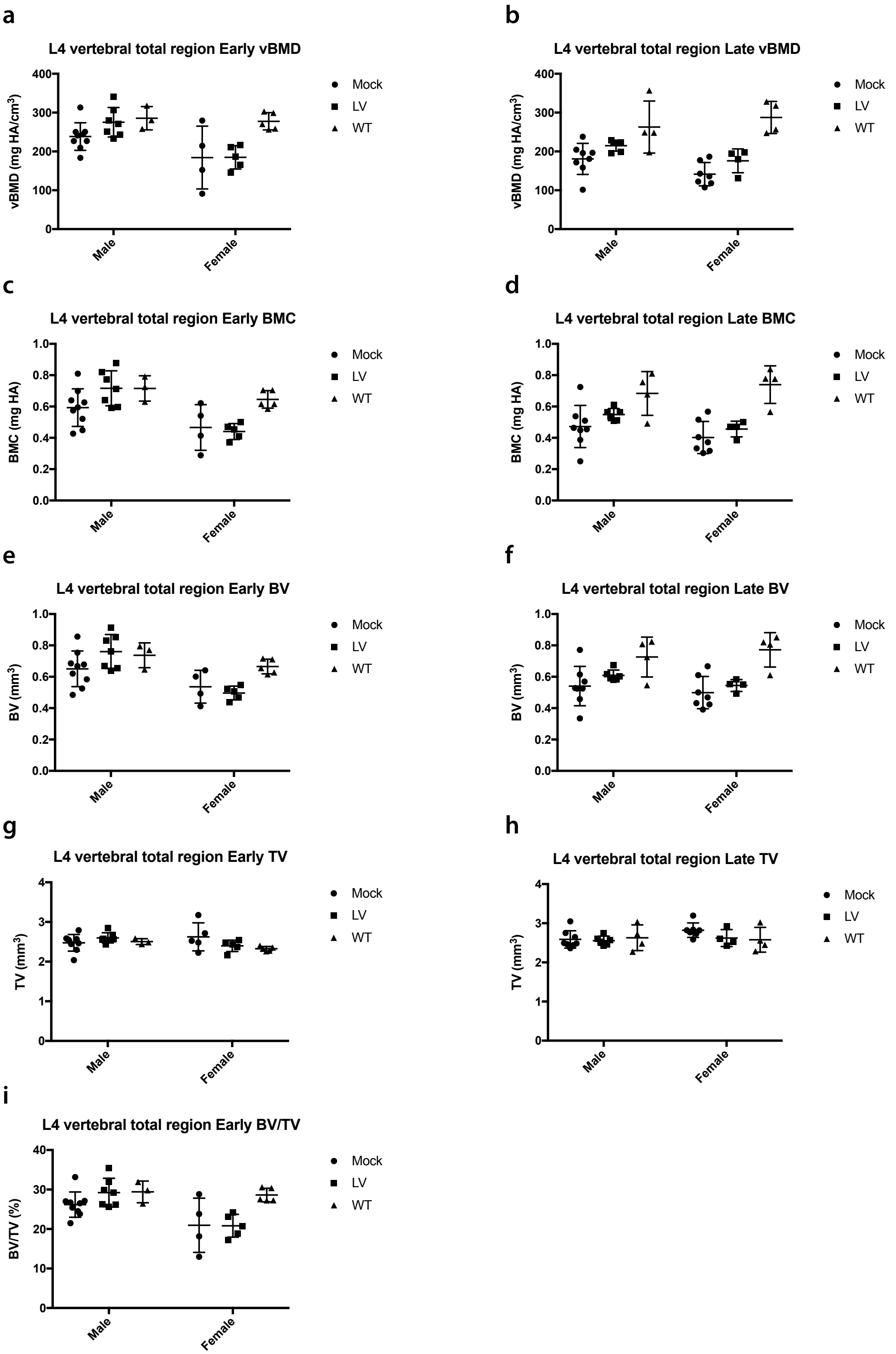

Fig S5) Micro-CT results from L4 vertebral region in male and female mice of Early and Late intervention study.

vBMD=volumetric bone mineral density, BMC=bone mineral content, BV=bone volume, TV=total volume, BV/TV=bone volume density. Early intervention samples in column to the left. Late intervention samples in column to the right.

Early intervention study males; Mock, *n=9*; LV, *n=8*; WT, *n=3*. Early intervention study females; Mock, *n=4*; LV, *n=5*; WT, *n=5*. Late intervention study males; Mock, *n=8*; LV, *n=6*; WT, *n=4*. Late intervention study females; Mock, *n=7*; LV, *n=4*; WT, *n=4*. Mann-Whitney *U*-test *p = ≤ 0.05; **p = ≤ 0.01; ***p = ≤ 0.001; **** = p = ≤ 0.0001. Error bars represent mean ± SD.

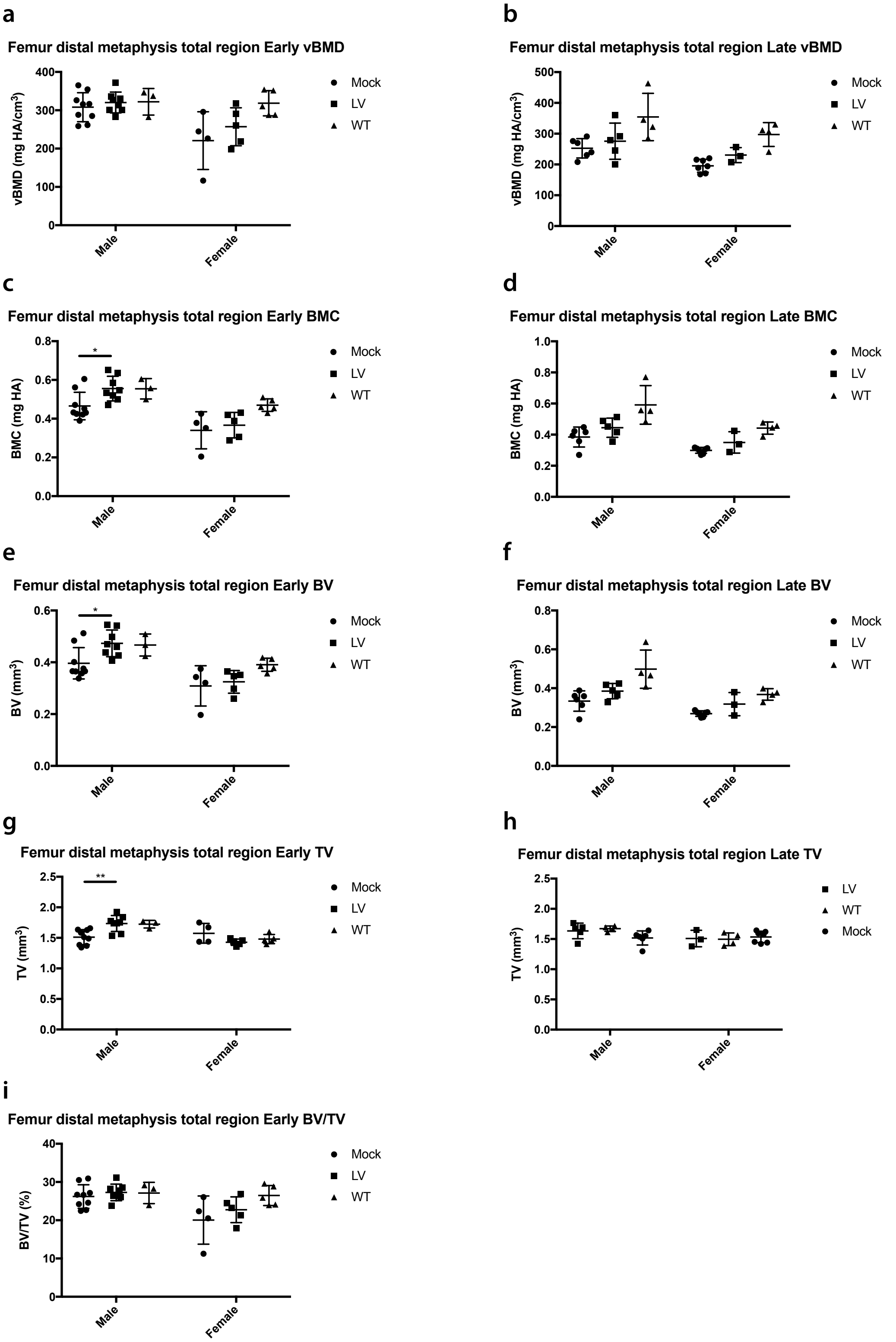

Fig S6) Micro-CT results from femur distal metaphysis region in male and female mice of Early and Late intervention study.

vBMD=volumetric bone mineral density, BMC=bone mineral content, BV=bone volume, TV=total volume, BV/TV=bone volume density. Early intervention samples in column to the left. Late intervention samples in column to the right.

Early intervention study males; Mock, *n=9*; LV, *n=8*; WT, *n=3*. Early intervention study females; Mock, *n=4*; LV, *n=5*; WT, *n=5*. Late intervention study males; Mock, *n=6*; LV, *n=5*; WT, *n=4*. Late intervention study females; Mock, *n=7*; LV, *n=3*; WT, *n=4.* Mann-Whitney *U*-test *p = ≤ 0.05; **p = ≤ 0.01; ***p = ≤ 0.001; **** = p = ≤ 0.0001. Error bars represent mean ± SD.

Table S1) Reoccurring insertions closest to individual genes (refseq_genes) between animals (overlaps) and for the *in vitro* control (multiple hit counts).

Between Animals	
**refseq_genes**	**overlap**
Lrrc4c	3
Ibtk	2

in vitro	
**refseq_genes**	**counts**
Lrrc4c	11
Mir101c	5
Tmem135	2
Celf4	2
Tmem114	2
Basp1	2
Lrtm1	2
Dcun1d5	2
Aplf	2
Ica1	2
Kit	2
Cdk5rap2	2
Fbxw7	2
Pcdh9	2
Loxl2	2
Arhgap10	2
